# The one‐carbon metabolic enzyme MTHFD2 promotes resection and homologous recombination after ionizing radiation

**DOI:** 10.1002/1878-0261.13645

**Published:** 2024-03-27

**Authors:** Petra Marttila, Nadilly Bonagas, Christina Chalkiadaki, Hannah Stigsdotter, Korbinian Schelzig, Jianyu Shen, Crystal M. Farhat, Amber Hondema, Julian Albers, Elisée Wiita, Azita Rasti, Ulrika Warpman Berglund, Ana Slipicevic, Oliver Mortusewicz, Thomas Helleday

**Affiliations:** ^1^ Science for Life Laboratory, Department of Oncology‐Pathology Karolinska Institutet Solna Sweden; ^2^ One‐carbon Therapeutics AB Stockholm Sweden; ^3^ Weston Park Cancer Centre, Department of Oncology and Metabolism, The Medical School University of Sheffield UK

**Keywords:** DSB repair, homologous recombination, ionizing radiation, MTHFD2

## Abstract

The one‐carbon metabolism enzyme bifunctional methylenetetrahydrofolate dehydrogenase/cyclohydrolase 2 (MTHFD2) is among the most overexpressed proteins across tumors and is widely recognized as a promising anticancer target. While MTHFD2 is mainly described as a mitochondrial protein, a new nuclear function is emerging. Here, we observe that nuclear MTHFD2 protein levels and association with chromatin increase following ionizing radiation (IR) in an ataxia telangiectasia mutated (ATM)‐ and DNA‐dependent protein kinase (DNA‐PK)‐dependent manner. Furthermore, repair of IR‐induced DNA double‐strand breaks (DSBs) is delayed upon *MTHFD2* knockdown, suggesting a role for MTHFD2 in DSB repair. In support of this, we observe impaired recruitment of replication protein A (RPA), reduced resection, decreased IR‐induced DNA repair protein RAD51 homolog 1 (RAD51) levels and impaired homologous recombination (HR) activity in *MTHFD2*‐depleted cells following IR. In conclusion, we identify a key role for MTHFD2 in HR repair and describe an interdependency between MTHFD2 and HR proficiency that could potentially be exploited for cancer therapy.

## Graphical abstract

In this study, we show that the MTHFD2 protein modulates homologous recombination (HR) repair of DNA double‐strand breaks (DSBs) in human cancer cells. After treatment with ionizing radiation, MTHFD2 is recruited, by ATM and DNA‐PK kinases, to the nucleus, where it promotes DNA end resection, and RPA and RAD51 loading during HR repair, thus contributing to cell survival.

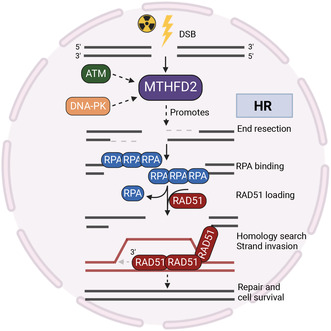

**Abbreviations**
ANOVA: analysis of variance
ATM: ataxia telangiectasia mutated
ATR: ataxia telangiectasia and Rad3‐related protein
BRCA1: breast cancer type 1 susceptibility protein
BRCA2: breast cancer type 2 susceptibility protein
BrdU: 5‐bromo‐2′‐deoxyuridine
DDR: DNA damage response
DNA‐PK: DNA‐dependent protein kinase
DSB: DNA double‐strand break
EdU: 5‐ethynyl‐2′‐deoxyuridine
HR: homologous recombination
iPOND: isolation of proteins on nascent DNA
IR: ionizing radiation
MTHFD1: methylene tetrahydrofolate dehydrogenase, cyclohydrolase and formyltetrahydrofolate synthetase 1
MTHFD2: bifunctional methylene tetrahydrofolate dehydrogenase/cyclohydrolase 2, mitochondrial
NHEJ: non‐homologous end joining
RAD51: DNA repair protein RAD51 homolog 1
RPA: replication protein A
ssDNA: single‐stranded DNA

## 1 Introduction

Maintaining genomic integrity is indispensable for the propagation of the intact genome to the next cell generation and cell survival. Therefore, considerable cellular resources are dedicated to coordinating DNA damage response (DDR) and repair of DNA damage caused by endogenous and exogenous sources, such as replication stress, carcinogens, and ionizing radiation. Among the different types of DNA lesions, DNA double‐strand breaks (DSBs) are considered the most harmful, as they can lead to chromosomal aberrations and trigger cell death if left unrepaired [1]. The ataxia telangiectasia mutated (ATM) kinase recognizes DSBs and is rapidly activated to coordinate the DDR signaling cascade [2, 3].

Activated ATM phosphorylates H2AX (γH2AX), which serves as a signal for the recruitment of various downstream DNA repair factors [4, 5]. DSBs are primarily repaired by either the non‐homologous end joining (NHEJ) or homologous recombination (HR) pathway [6]. The choice between NHEJ or HR depends on cell cycle stage and how the dsDNA end is processed. During HR, the MRN‐complex mediates DNA end resection, which depends on the activity of nucleases to create single‐stranded DNA (ssDNA) overhangs needed for homology search and strand pairing [7]. The replication protein A (RPA) rapidly binds and stabilizes ssDNA but is subsequently replaced by RAD51 in a breast cancer susceptibility 1 and 2 protein (BRCA1/2)‐dependent process [8], which catalyzes strand invasion and enables DNA synthesis and repair [9, 10, 11].

The bifunctional folate enzyme methylene tetrahydrofolate dehydrogenase/cyclohydrolase 2 (MTHFD2) is widely overexpressed in cancer, possesses a highly cancer‐specific expression profile, and has been validated extensively as a promising anticancer target in various tumor types [12, 13, 14, 15, 16, 17, 18, 19, 20, 21, 22, 23]. While its role in mitochondrial one‐carbon folate metabolism is well characterized, recent findings have described additional roles for MTHFD2 in the nucleus, particularly in relation to RNA processing and DNA replication [14, 24, 25]. Furthermore, MTHFD2 has been ascribed a non‐catalytic role in regulating PARP3‐dependent end joining, specifically in p53‐defective cells [26]. *Mthfd2* knockdown has also been shown to impair RAD51 foci formation upon camptothecin treatment in mouse embryonic stem cells, and MTHFD2 has been suggested to promote HR by binding CDK1 and EXO1 [27]. Altogether, this points to a potential role of MTHFD2 in DNA repair, which may be therapeutically relevant.

Here we show that ionizing radiation (IR) leads to enhanced nuclear accumulation and chromatin association of MTHFD2. Depletion of *MTHFD2* abolishes IR‐induced recruitment of RPA and RAD51 into repair foci, blocks HR activity and ultimately results in a high amount of unresolved DSBs and accumulation of cancer cells in G2/M phase.

## 2 Materials and methods

### 2.1 Cell culture

U2OS osteosarcoma (HTB‐96, RRID:CVCL_0042), HCT116 colorectal cancer (CLL‐247, RRID:CVCL_0291) and SW620 colorectal cancer (CCL‐227, RRID:CVCL_0547) were obtained from ATCC (Manassas, VA, USA). SW620 *MTHFD2* ^−/−^ cells were purchased from Synthego (Redwood City, CA, USA) using single‐guide RNA sequences targeting exon 2 (5′‐CGCCAACCAGGAUCACACUC‐3′ for *MTHFD2* ^−/−^) [28]. All cell lines were cultured in RPMI GlutaMAX (Thermo Fisher Scientific, Waltham, MA, USA), supplemented with 10% heat‐inactivated FBS (Thermo Fisher Scientific), 100 U·mL^−1^ penicillin and 100 μg·mL^−1^ streptomycin (Thermo Fisher Scientific). Cell lines were authenticated within the past 3 years using short‐tandem repeat analysis. Cell lines were maintained at 37 °C in 5% CO_2_ humidified incubators, and routinely tested for mycoplasma using MycoAlert™ Mycoplasma Detection Kit (Lonza, Basel, Switzerland).

### 2.2 Small‐molecule inhibitors

The following commercial compounds were used: Etoposide (#341205; Sigma Aldrich, St. Louis, MO, USA), ATM inhibitor KU‐55933 (#3544; Tocris, Bristol, UK), DNA‐PK inhibitor NU‐7441 (#3712; Tocris), ATR inhibitor VE‐821 (#HY‐14731; MedChem Express, Monmouth Junction, NJ, USA).

### 2.3 Ionizing radiation

γ‐irradiation was performed using a ^137^Cs source (Scanditronix, Uppsala, Sweden) at a photon dose rate of 0.5 Gy·min^−1^. X‐ray high‐intensity radiation was performed with an X‐RAD 225 XL irradiator (Precision X‐Ray, Madison, CT, USA) set with a 2 mm aluminum filter. Cells treated with either γ‐irradiation or X‐rays were exposed to comparable doses equivalent to 1, 2, or 5 Gy X‐ray radiation.

### 2.4 RNAi knockdown

Unless otherwise specified, depletion of endogenous *MTHFD2* was achieved using a pool of two different ON‐TARGETplus MTHFD2 siRNA oligonucleotides (#1: D‐009349‐02 and #2: J‐009349‐12; Dharmacon™, Horizon Discovery, Waterbeach, UK). *MTHFD1* was depleted using a SMARTPool of ON‐TARGETplus human MTHFD1 siRNA (L‐009577‐00‐0005; Dharmacon™, Horizon Discovery) *RAD51* was depleted using with the siRNA oligonucleotide sequence GGGAAUUAGUGAAGCCAAATT (SI02663682; Qiagen, Hilden, Germany). ON‐TARGETplus non‐targeting siRNA pool was used as control (D‐001810‐10‐20; Dharmacon™, Horizon Discovery). Transfection was performed on log‐phase cells using 10 nm siRNA and INTERFERin® transfection reagent (Polyplus‐Transfection, Illkirch, France), following the manufacturer's protocol. Cells were incubated with the siRNAs for 24 h, if not otherwise specified, at 37 °C in a humidified incubator with 5% CO_2_ until irradiation and harvesting.

### 2.5 Isolation of proteins on nascent DNA (iPOND)

iPOND was performed as described previously [29]. Briefly, 10 million SW620 cells per dish were seeded in 10 150 mm culture dishes and let grow for 3 days to reach confluency of approximately 80% before pulsing with 10 μm 5‐ethynyl‐2′‐deoxyuridine (EdU, Sigma Aldrich) for 20 min. For the chase samples, after the EdU labelling, 10 μm thymidine was added for additional 2 h. After treatment, cells were cross‐linked with 1% formaldehyde in PBS for 20 min and quenched with 125 mm glycine (Sigma Aldrich) for 5 min at room temperature. Then, cells were scraped, washed tree times with PBS at 4 °C and permeabilized with 0.25% Triton X‐100 in PBS (10^7^ cells·mL^−1^) for 30 min at room temperature. Cells were first washed with 0.5% BSA in PBS, followed by a second wash in PBS. Cells were then resuspended in the Click‐IT reaction cocktail [10 μm biotin‐azide (Thermo Fisher Scientific), 10 mm sodium ascorbate, 2 mm CuSO_4_ in PBS] at a concentration of 2 × 10^7^ cells·mL^−1^ and incubated for 1 h at RT under continuous rotation. For the negative control, biotin‐azide was omitted from the Click‐IT reaction. Cells were centrifuged at 1000 ***g*** for 5 min at 4 °C and washed twice, first with 0.5% BSA in PBS and then in PBS. The pellet was resuspended in lysis buffer (50 mm Tris–HCl pH 8.0, 1% SDS, 1× protease inhibitors) at a concentration of 1.5 × 10^7^ cells per 100 μL and lysed for 10 min, followed by sonication (four 10 s ON/10 s OFF cycles, amplitude 50%). Samples were centrifuged at 17 000 ***g*** for 30 min at 4 °C and the lysate was cleared by passing through a 0.45 μm PVDF filter (Merck Millipore, Burlington, MA, USA). Samples were diluted 1 : 1 (v/v) with cold PBS containing 1× protease inhibitors and 20 μL of lysate was saved as input. Samples were then immunoprecipitated with streptavidin‐agarose beads (Novagen®, Merck Millipore) overnight at 4 °C. Beads were washed three times for 5 min at room temperature with 1 mL of lysis buffer and boiled with 2× SDS sample buffer (Bio‐Rad, Hercules, CA, USA) with 200 mm DTT (Merck Millipore) for 25 min. Boiled samples were centrifuged at 2500 ***g*** for 1 min at room temperature, separated on 4–15% gradient gel and analyzed by western blotting.

### 2.6 Western blot

Cells pellets were incubated for 30 min on ice in lysis buffer [100 mm Tris–HCl, pH 8.0, 150 mm NaCl, 1% NP‐40, 1× cOmplete protease inhibitor cocktail (Roche, Basel, Switzerland) and 1× Halt phosphatase inhibitor cocktail (Thermo Fisher Scientific)] and sonicated. Lysates were centrifuged at 25 000 ***g***, 20 min at °C. Supernatant was collected and stored at −80 °C. Protein concentration was determined using the Pierce BCA Protein Assay Kit (Thermo Fisher Scientific). Proteins were loaded on 4–15% PROTEAN TGX precast cells (Bio‐Rad) using 4× NuPage LDS sample buffer (Thermo Fisher Scientific) and blotted onto nitrocellulose membrane (Bio‐Rad) according to standard protocols. Membranes were blocked with 5% milk in TBS Tween‐20 (TBS‐T, Thermo Fisher Scientific) or Intercept blocking buffer (LI‐COR Biosciences, Lincoln, NE, USA) diluted 1 : 1 in TBS‐T Tween‐20 and incubated with primary antibodies overnight at 4 °C. Incubation with secondary antibody was performed for 1 h at room temperature. Detection was done with Odyssey Fc imager (LI‐COR Biosciences). imagestudiolite v.5.2.5 (LI‐COR Biosciences) and imagej (NIH, Bethesda, MD, USA) were used for analysis. Uncropped immunoblots are shown in the Supporting Information.

### 2.7 Subcellular fractionation

Cells were collected and washed in ice‐cold PBS, resuspended ice‐cold hypotonic buffer (10 mm HEPES pH 7.5, 10 mm KCl, 100 nm EDTA, 100 nm EGTA, 1 mm DTT, protease and phosphatase inhibitors) for 15 min, then lysed in 1% NP‐40 and centrifuged 5 min at 3000 ***g***. The supernatant was collected as the cytoplasmic fraction. When isolating only chromatin‐bound proteins, nuclear pellet was washed twice in hypotonic buffer and spun down at 3000 ***g*** for 5 min at 4 °C. Chromatin‐bound extraction buffer was prepared by adding 2.5 μL 10× MNase buffer, 0.25 μL 100× BSA, and 1 μL Micrococcal Nuclease (New England Biolabs, Ipswich, MA, USA) to 25 μL nuclease buffer (150 mm NaCl, 5 mm MgCl_2_) at RT. Pellets were resuspended in nuclease extraction buffer, vortexed for 15 s, incubated for 10 min at 37 °C, and vortexed for 15 s, and finally centrifuged at 13 000 ***g*** for 10 min. Western blot was performed as described above.

### 2.8 Immunoprecipitations

U2OS cells were seeded in 150 mm dishes at a density of 1.5 million cells per dish and let grow for 72 h before the cells were exposed to IR (5 Gy) or left untreated. Two hours after IR, cells were incubated in cold lysis buffer (50 mm HEPES pH 7.4, 150 mm NaCl, 1 mm EDTA, 1 mm EGTA, 10% glycerol, 1% Triton X‐100, 1× protease inhibitors, 1× phosphatase inhibitors) for 15 min and harvested by scraping. Lysates were cleared by centrifuging for 20 min at 16 000 ***g***, 4 °C. Protein concentration was determined using Pierce™ 660 nm Protein Assay Reagent (Thermo Fisher Scientific) according to the manufacturer's instructions, and 500 μg of total protein was then immunoprecipitated with 2 μg of either mouse IgG control (Thermo Fisher Scientific) or mouse anti‐MTHFD2 primary antibody (56772; Abcam, Cambridge, UK) overnight at 4 °C on a rotating wheel. The following day, 40 μL of Dynabeads Protein A slurry was added for 2 h at 4 °C, then protein complexes were washed four times in ice‐cold 500 μL of lysis buffer and finally eluted by adding 60 μL of NuPage LDS sample buffer supplemented with NuPage reducing agent and boiling at 70 °C for 10 min. Proteins were separated by SDS/PAGE followed by western blotting.

### 2.9 Immunofluorescence microscopy

Fifty thousand cells were seeded in 12‐well plates on sterilized glass coverslips (VWR) and left to attach overnight prior to treatment. Cells were then washed with PBS and fixed in 4% paraformaldehyde (PFA, Santa Cruz Biotechnology, Dallas, TX, USA) and 4% sucrose in PBS for 15 min at room temperature. Cells were permeabilized using 0.2% NP‐40 (Thermo Fisher Scientific) in PBS for 10 min, then incubated with blocking buffer (2% BSA/0.2% NP‐40/PBS) for 30 min at room temperature. Coverslips were placed in a humidified chamber with primary antibodies in antibody buffer (3% BSA/0.1% NP‐40/PBS) at 4 °C overnight, gently rinsed 3× in PBS, then incubated with secondary antibodies diluted in the antibody buffer for 45 min at room temperature, followed by DNA staining with DAPI (Sigma Aldrich) for 10 min. Coverslips were mounted with ProLong Gold mounting medium (Thermo Fisher Scientific) and allowed to cure 24 h at room temperature on a flat surface away from light. Images were acquired with a Zeiss LSM 780 confocal microscope using a 40× immersion oil objective. Image analysis was performed with imagej (NIH) and cellprofiler (Broad Institute, Cambridge, MA, USA) software.

### 2.10 *In situ* cell fractionation

To visualize BrdU, RPA32 and RPA70 foci, the cells were first washed with PBS and then incubated for 3 min at room temperature with cytoskeleton (CSK) buffer containing 10 mm PIPES pH 7.0 (Sigma Aldrich), 100 mm NaCl (VWR), 300 mm sucrose (Sigma Aldrich), 3 mm MgCl_2_ (Sigma Aldrich), and 0.7% Triton X‐100 (Sigma Aldrich). Cells were then washed once again with PBS and fixed in 4% PFA/2% sucrose in PBS.

### 2.11 BrdU labelling of cultured cells

U2OS cells were seeded on sterilized glass coverslips, let attach overnight and transfected with control or MTHFD2 siRNA for 24 h. Cells were then washed once with warm media before adding fresh media with 10 μm 5‐bromo‐2′‐deoxyuridine (BrdU, Sigma Aldrich) and incubated for additional 24 h to label genomic DNA. Thereafter, cells were irradiated at 2 Gy and let to recover for 2 h before *in situ* extraction with CSK buffer and fixation with 4% PFA/2% sucrose in PBS. Immunofluorescence staining was performed as described above.

### 2.12 Antibodies

The following antibodies were used for western blotting (WB), immunofluorescence (IF) and immunoprecipitation (IP): rabbit anti‐MTHFD1 (Atlas Antibodies, Bromma, Sweden, HPA000704, WB: 1000, IF 1 : 500), mouse anti‐MTHFD2 (Abcam, ab56772, WB 1 : 500, IF 1 : 100, IP 2 μg), rabbit anti‐MTHFD2 (Cell Signaling Technology, Danvers, MA, USA, 41377, WB 1 : 1000), rabbit anti‐PCNA (Abcam, ab18197, WB 1 : 1000), rabbit anti‐Histone H3 (Abcam, ab1791, WB 1 : 20 000), mouse anti‐α‐Tubulin (Abcam, ab7291, WB 1 : 20 000), rabbit anti‐β‐Tubulin (Abcam, ab6046, WB 1 : 1000), mouse β‐actin (Abcam, ab6276, WB 1 : 10 000), rabbit anti‐COX IV (Proteintech, Rosemont, IL, USA, 11242‐1‐AP, WB 1 : 2000), rabbit anti‐phospho‐ATM S1981 (Abcam, ab81292, WB 1 : 500), rabbit ATM (D2E2) (Cell Signaling Technology, 2873, WB 1 : 1000), rabbit DNA‐PKcs (Cell Signaling Technology, 4602, WB 1 : 1000), mouse anti‐ɣH2AX S139 (Millipore, 05‐636WB, WB 1 : 500, IF 1 : 250), rabbit anti‐ɣH2AX S139 (Cell Signaling Technology, 2577, IF 1 : 250), rat anti‐RPA32 (Cell Signaling Technology, 2208, IF 1 : 250), rabbit RPA70 (Cell Signaling Technology, 2267, WB 1 : 1000, IF 1 : 250), rabbit anti‐RAD51 H‐92 (Santa Cruz Biotechnology, sc‐8349, WB 1 : 500), rabbit anti‐RAD51 (Abcam, ab63801, IF 1 : 500), rabbit p‐BRCA1 S1524 (Cell Signaling Technology, 9009, WB 1 : 500), mouse anti‐BrdU (BD Biosciences, Franklin Lakes, NJ, USA, 347580, IF 1 : 300).

Secondary antibodies used for western blotting were IRDye 800CW Donkey Anti‐Mouse IgG (H + L) (Li‐Cor Biosciences, 926‐32212; 1 : 5000), IRDye 800CW Donkey Anti‐Rabbit IgG (H + L) (Li‐Cor Biosciences, 926‐32213; 1 : 5000), Peroxidase AffiniPure Donkey Anti‐Rabbit IgG (Jackson ImmunoResearch, West Grove, PA, USA, 711‐035‐152; 1 : 10 000) and Peroxidase AffiniPure Donkey Anti‐Mouse IgG (Jackson ImmunoResearch, 715‐035‐150; 1 : 10 000).

Secondary antibodies used for immunofluorescence were: goat anti‐rat IgG Alexa Flour 568 (Thermo Fisher Scientific, A‐11077, 1 : 500), donkey anti‐mouse IgG Alexa Fluor 568 (Thermo Fisher Scientific, A‐10037, 1 : 500), goat anti‐rabbit IgG Alexa Fluor 647 (Thermo Fisher Scientific, A‐21245, 1 : 500) and donkey anti‐rabbit IgG Alexa Fluor 647 (Thermo Fisher Scientific, A‐31573, 1 : 500).

### 2.13 Clonogenic survival assay

Cells were transfected with indicated siRNAs and 24 h later exposed to IR (1, 2 or 5 Gy) or left untreated and allowed to recover for 4 h before re‐seeding 500 U2OS or 200 HCT116 cells in 6‐well plates. After 10–14 days colonies were fixed and stained with 4% methylene blue (Sigma Aldrich) in methanol for 20 min at room temperature. Colonies were manually counted and normalized to plating efficiencies of non‐irradiated siCtrl. Raw image data on representative colonies is shown in the Supporting Information.

### 2.14 Cell growth assay

Cells were transfected with indicated siRNAs for 24 h, exposed to IR (2 Gy) or left untreated and allowed to recover for 4 h before re‐seeding 3000 U2OS cells or 1500 HCT116 cells in 96‐well plates. For studies with CRISPR‐Cas9 *MTHFD2* knockout cells, 4000 SW620 wildtype or *MTHFD2* ^−/−^ cells were seeded. Cell confluency was recorded every 24 h during a period of 5–7 days by acquiring brightfield images of the wells with Tecan Spark Cyto plate reader (Tecan, Männedorf, Switzerland) using 4× magnification. Calculated confluency values (percentage surface area covered by the cells) for each time point were plotted in graphpad prism software to establish growth curves.

### 2.15 HR repair assay (DR‐GFP)

HR repair assay was performed in U2OS (DR‐GFP) cells as previously described [30].

### 2.16 Flow cytometry cell cycle and DNA damage analysis

Cells were collected, washed, and fixed in PFA 4% solution for 15 min. Samples were washed in ice‐cold BSA buffer (1% BSA/PBS) and permeabilized in 300 μL saponin buffer (0.1% saponin, 1% BSA/PBS) for 30 min on ice. Cells were incubated in 50 μL primary antibody solution (mouse α‐γH2AX, Millipore 05‐636, 1 : 1000 in saponin buffer) overnight at 4 °C. Samples were washed in ice‐cold BSA buffer, then resuspended in 50 μL secondary antibody solution (donkey α‐mouse Alexa Fluor 647, InvitrogenTM, Thermo Fisher Scientific, A31571, 1 : 50 in saponin buffer) and incubated 30 min at 37 °C. After washing in ice‐cold BSA buffer, cells were resuspended in 300 μL of Hoechst staining buffer (10 μg·mL^−1^ Hoechst, 100 μg·mL^−1^ RNase A in BSA buffer), incubated for 15 min at room temperature, and analyzed using a Navios flow cytometer (Beckman Coulter, Brea, CA, USA). Analysis of flow cytometry data was performed using kaluza software (Beckman Coulter). Gating strategy was determined by unstained and single‐stain controls, together with etoposide‐treated positive DNA damage control cells. At least 10 000 events were acquired per sample. Collected data were processed using kaluza software v1.3 (Beckman Coulter) and analyzed using a one‐way ANOVA with Tukey's correction for multiple comparisons using prism v8.0 (GraphPad Software, La Jolla, CA, USA).

### 2.17 Quantification and statistical analysis

All data were plotted and statistical analysis was carried out in prism v.9.5.0 (GraphPad Software). Data are plotted as means ± standard deviation (SD) as indicated in the figure legends. Standard deviation is indicated for all cases where *n* ≥ 3. The number of replicates (*n*) corresponds to the number of independent experiments, individual cells or nuclei in the case of single‐cell data or independent cell cultures and is indicated in the figure legends.

## 3 Results

### 3.1 Association of MTHFD2 with chromatin in the nucleus is not restricted to replication

**Fig. 1:**
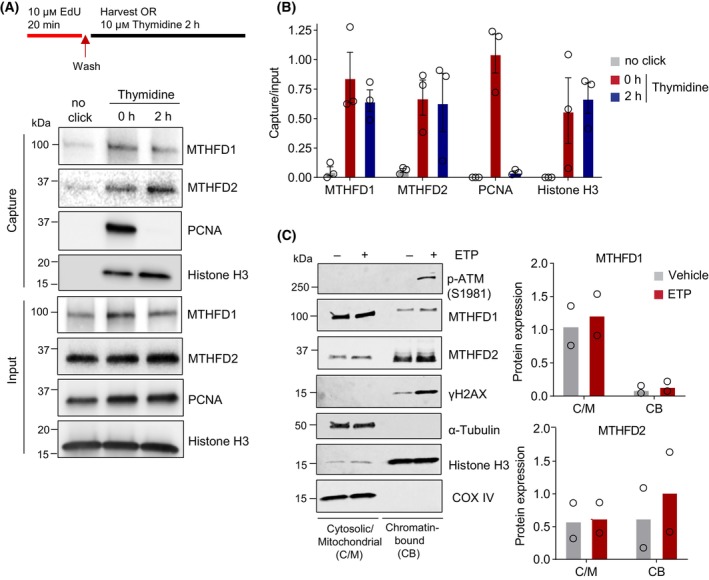
Association of MTHFD2 with chromatin in the nucleus is not restricted to replication. (A) Isolation of proteins on nascent DNA (iPOND) analysis of MTHFD1 and MTHFD2 at replication forks. SW620 cells were first pulse‐labeled with 10 μm EdU, followed by a chase with 10 μm thymidine for 2 h to distinguish between true replication proteins. Shown are representative western blots of input and iPOND purified proteins of *n* = 3 independent experiments. PCNA and Histone H3 act as experimental controls. (B) Gel densitometric quantification of MTHFD1, MTHFD2, PCNA and Histone H3 from (A). Capture signals were normalized to their respective inputs. Data are displayed as means ± SEM, *n* = 3 independent experiments. (C) Left, SW620 cells treated with vehicle of 10 μm etoposide (ETP) for 4 h were subjected to cell fractionations for isolation of cytosolic and mitochondrial (C/M), and chromatin‐bound (CB) proteins, followed by immunoblotting. Shown are representative blots of *n* = 2 independent experiments. Right panel depicts gel densitometric quantification of MTHFD1 and MTHFD2 protein levels in the different fractions. Signals from the C/M fractions were normalized to their respective α‐Tubulin signal, and signals from the CB fractions were normalized to their respective Histone H3 signal. Bar graphs display the mean of normalized protein signal.

While the MTHFD2 protein is a known mitochondrial enzyme required for formate release and cell growth, a novel role in the nucleus associated with replication forks has been reported [24]. To test the association with nascent replication forks, we performed the iPOND assay [29] (Fig. 1A) and also included a chase period in order to determine if the protein is also associated with post‐replicative DNA beyond the active replication fork. Here, we found that the PCNA protein is, as expected, associated only with nascent replication forks, while the MTHFD2 protein is also associating with DNA beyond nascent replication forks, similar to Histone H3 (Fig. 1A,B). Interestingly, we also investigated the cytosolic isoenzyme MTHFD1 and found that this is also associated with DNA, irrespective of replication, thus confirming the previous observations of MTHFD1 being recruited to chromatin [31].

Next, we treated cells with the topoisomerase II inhibitor etoposide (VP‐16), which induces DSBs, preferentially at replication forks [32], and found a slight increase in chromatin association with the MTHFD2 protein, but not with MTHFD1 (Fig. 1C), suggesting MTHFD2 could be involved in response to DNA damage, in line with previous papers indicating a role for MTHFD2 in DNA repair [26, 27]. Altogether, these data suggest that MTHFD2 is not a part of the replication process itself, but is potentially recruited to damaged replication forks.

### 3.2 MTHFD2 accumulates in the nucleus following ionizing radiation

**Fig. 2:**
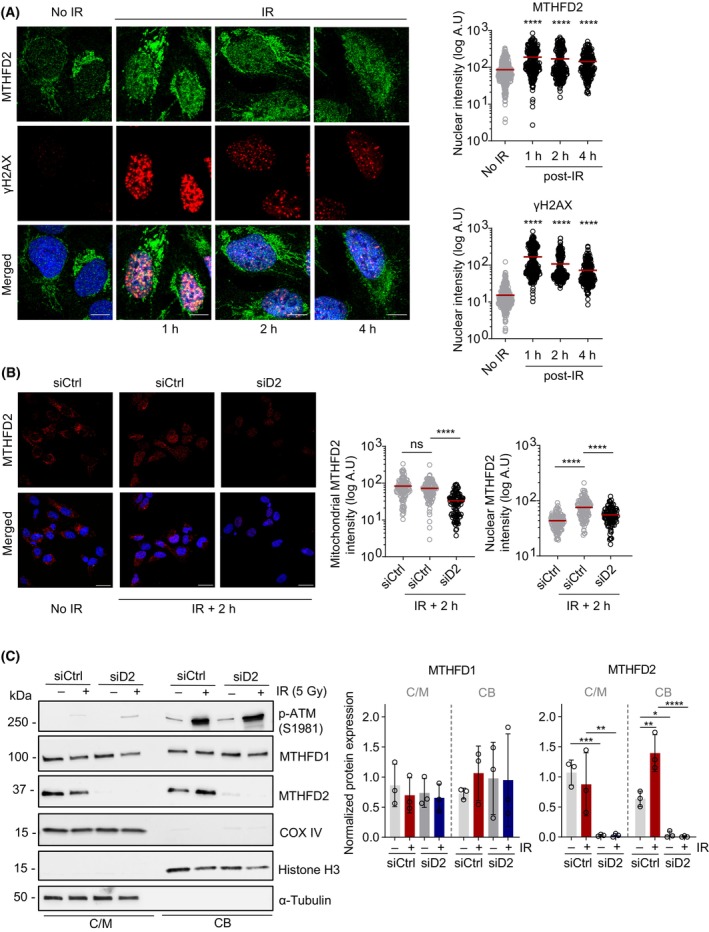
MTHFD2 accumulates in the nucleus following ionizing radiation. (A) Confocal analysis of localization of MTHFD2 and induction γH2AX in U2OS cells subjected to irradiation (5 Gy) and fixed at indicated time points post‐irradiation (IR). Shown is one out of two independent experiments. To the right, scatter dot plots show nuclear MTHFD2 and ɣH2AX intensities per nuclei. Red bars represent mean intensities per nucleus, n (nuclei) = 286 (no IR), 245 (1 h post‐IR), 232 (2 h post‐IR) and 213 (4 h post‐IR). ****P < 0.0001; Kruskal–Wallis test with Dunn's multiple comparisons test. Scale bar on representative images, 10 μm. (B) Confocal analysis of mitochondrial and nuclear localization of MTHFD2 in U2OS cells following treatment with siCtrl or siMTHFD2 (siD2) at 10 nm for 24 h and subjected to irradiation (5 Gy, 2 h recovery), or left untreated. Shown is one out of three independent experiments. Scale bar, 20 μm. To the right, scatter dot plots showing MTHFD2 intensities as quantified using cellprofiler (Broad Institute). Red bars indicate the mean intensity, n (cells) = 117 (siCtrl, no IR), 114 (siCtrl, IR + 2 h) and 102 (siMTHFD2, IR + 2 h). ns, not significant with *P* > 0.05, ****P < 0.0001; Kruskal–Wallis test with Dunn's multiple comparisons test. (C) U2OS cells treated with siCtrl or siMTHFD2 and exposed to IR (5 Gy, 2 h recovery) or left untreated were subjected to cell fractionations for isolation of cytosolic and mitochondrial (C/M), and chromatin‐bound (CB) proteins, followed by immunoblotting. Shown are representative blots of *n* = 3 independent experiments. To the right, gel densitometric quantification of MTHFD1 and MTHFD2 protein levels in the different fractions. Bands from the C/M fractions were normalized to their respective α‐tubulin signal, and bands from the CB fractions were normalized to their respective Histone H3 signal. Bar graphs display the mean ± SD of normalized protein signal. *P < 0.05, **P < 0.01, ***P < 0.001, ****P < 0.0001; one‐way ANOVA with Šídák's multiple comparisons test.

While DSBs formed following treatment with etoposide preferentially occur at replication forks, ionizing radiation (IR)‐induced DSBs are less restricted to the S phase of the cell cycle [32, 33]. To test if MTHFD2 is involved in DNA repair processes following IR, we treated cells with a 5 Gy dose of IR and examined the subcellular localization of MTHFD2 after the treatment. Interestingly, we could observe a marked accumulation of MTHFD2 protein already 1 h after irradiation (Fig. 2A), particularly in the nucleus, indicating that MTHFD2 responds and translocates into the nucleus after IR‐induced DSBs. In support of this, we can see that the induction of MTHFD2 in the nucleus correlates with induction of γH2Ax foci formation (Fig. S1A,B). This rapid accumulation of MTHFD2 in the nucleus suggests that it contributes to an early IR response.

In cells treated with siRNA targeting *MTHFD2* we could detect an overall reduction of immunofluorescence staining of MTHFD2 protein in the mitochondria and in the nucleus after IR. This supports the notion that the antibody recognizes MTHFD2 and that the increased nuclear staining with the antibody represents presence of the MTHFD2 protein (Fig. 2B and Fig. S1C). Moreover, using western blotting we could confirm a statistically significant increase in MTHFD2 protein levels in the chromatin‐bound fraction following IR treatment (Fig. 2C). We also performed these experiments in *MTHFD2*‐depleted cells and found markedly reduced levels of MTHFD2 staining and no accumulation of MTHFD2 in the chromatin‐bound fraction after IR. These experiments further strengthen the notion that MTHFD2 is translocated to the nucleus and responds to DNA damage upon IR treatment.

While a fraction of MTHFD1 also resides at the chromatin (Fig. 1A,C), we found no evidence of altered chromatin recruitment of MTHFD1 using etoposide (Fig. 1C). Upon IR treatment, we could not detect any statistically significant changes in chromatin‐bound MTHFD1 protein using western blotting (Fig. 2C). Similarly, we found no evidence of nuclear accumulation of MTHFD1 in the nucleus following IR treatment in our immunofluorescence assays, but observed a slight decrease in nuclear MTHFD1 levels instead (Fig. S1D,E). This suggests that MTHFD2, but not MTHFD1, may be more prominently involved in the IR response.

**Fig. 3:**
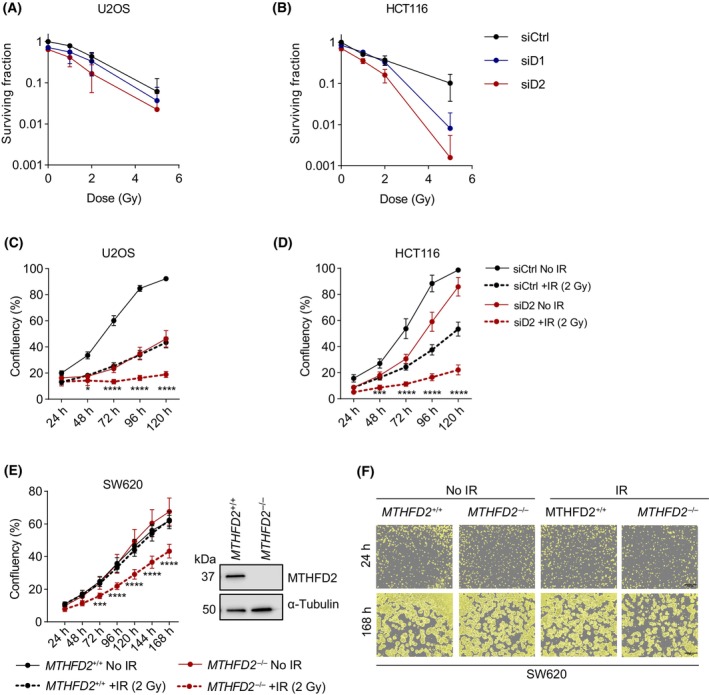
Knockdown of *MTHFD2* impairs cell survival and proliferation following IR. (A, B) Clonogenic survival assay of U2OS (A) and HCT116 (B) cells upon siRNA depletion (24 h) of *MTHFD1* (siD1) or *MTHFD2* (siD2), followed by irradiation (IR) at indicated doses. Colonies were stained with 4% methanol blue in methanol, counted and normalized to the non‐irradiated siCtrl. Data are pooled from two independent experiments performed in triplicate and displayed means ± SD, *n* = 6 independent cell cultures. (C, D) Cell proliferation assay of U2OS (C) and HCT116 (D) cells upon siRNA depletion (24 h) of *MTHFD2* (siD2), followed by irradiation at 2 Gy or left untreated. Percentage confluency (determined by the occupied area) was measured every 24 h after irradiation. Shown is one out of three independent experiments. Data are displayed as means ± SD, *n* = 10 independent cell cultures. Statistical significance is reported for comparisons between irradiated siCtrl and irradiated siD2 groups. *P < 0.05, ***P < 0.001, ****P < 0.0001; one‐way ANOVA with Šídák's multiple comparisons test. (E) SW620 wildtype (*MTHFD2* ^+/+^) and SW620 *MTHFD2* ^−/−^ cells were irradiated at 2 Gy or left untreated and cell proliferation (percentage confluency) was measured every 24 h after irradiation. Shown is one out of two independent experiments. Data are displayed as means ± SD, *n* = 10 independent cell cultures. Statistical significance is reported for comparisons between irradiated wildtype and irradiated *MTHFD2* ^−/−^ groups. ***P < 0.001, ****P < 0.0001; one‐way ANOVA with Šídák's multiple comparisons test. To the right, a western blot verification of MTHFD2 knockout in SW620 cells. (F) Representative images of fields used to calculate confluency reported in (E). Shown are representative wells for 24 and 168 h time points, *n* = 2 independent experiments. Images were acquired with Tecan Spark Cyto plate reader (Tecan; brightfield microscope, 4× magnification). Scale bar, 500 μm.

Next, we wanted to determine if the IR‐induced increase in MTHFD2 levels is related to cellular survival. Using siRNA targeting, we could observe that *MTHFD2* depletion consistently reduced clonogenic survival in both U2OS and HCT116 cell lines (Fig. 3A,B and Fig. S2). This was also consistent with reduced growth rates following *MTHFD2* siRNA depletion in both cell lines (Fig. 3C,D and Fig. S3). Interestingly, we also found significantly reduced proliferation in CRISPR‐Cas9‐generated *MTHFD2* ^−/−^ SW620 cells after IR treatment (2 Gy) as compared to the relatively radioresistant wildtype cells (Fig. 3E,F). Taken together, these data indicate a potential role of MTHFD2 in the repair of IR‐induced DNA damage.

### 3.3 ATM and DNA‐PK mediate MTHFD2 protein accumulation in the nucleus following induction of DSBs

**Fig. 4:**
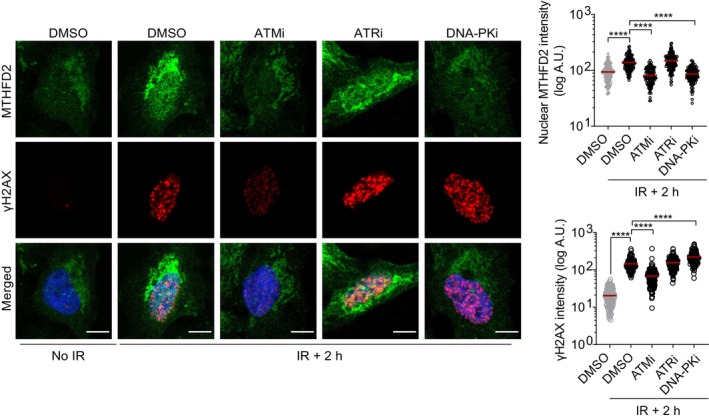
ATM and DNA‐PK mediate MTHFD2 protein accumulation in the nucleus following induction of double‐strand breaks (DSBs). Confocal analysis of subcellular MTHFD2 levels in U2OS cells 2 h post‐ionizing radiation (IR 5 Gy), following a 6 h pre‐incubation with ATMi (KU‐55933, 10 μm), ATRi (VE‐821, 2.5 μm), DNA‐PKi (NU‐7441, 2 μm) or DMSO control. Shown is one out of three independent experiments. Scale bar on representative images, 10 μm. To the right, quantification of mean nuclear MTHFD2 and γH2AX intensities. Red bars represent mean intensities per nucleus, n (nuclei) = 124 (DMSO no IR), 130 (DMSO IR + 2 h), 119 (ATMi IR + 2 h), 144 (ATRi IR + 2 h) and 118 (DNA‐PKi IR + 2 h). ****P < 0.0001; one‐way ANOVA analysis with Tukey's multiple comparisons test.

To narrow down the DNA repair pathways in which MTHFD2 could be involved, we separately inhibited the three main DDR kinases, ataxia telangiectasia mutated (ATM), ATM‐ and Rad3‐related (ATR) and the DNA‐dependent protein kinase (DNA‐PK) prior to IR [34], and analyzed their effect on MTHFD2 nuclear accumulation in U2OS cells. While inhibition of ATR did not affect MTHFD2 nuclear levels upon IR, it was abolished by both ATM and DNA‐PK inhibition (Fig. 4), consistent with ATM being the main DDR kinase involved in response to IR‐induced DSBs [35] and DNA‐PK being the main kinase activated at DSBs to mediate NHEJ repair [36]. Collectively, these data show that, upon IR, ATM‐mediated signaling and DNA‐PK activity are required to induce rapid and sustained accumulation of MTHFD2 in the nucleus. As nuclear recruitment of MTHFD2 was dependent on both the DNA‐PK and ATM, we investigated if this is mediated by binding between MTHFD2 and ATM or DNA‐PK. By using co‐IP experiments, we could not find evidence of MTHFD2 binding to either ATM or DNA‐PK with or without IR treatment (Fig. S4A). Moreover, genome‐wide phosphorylation mapping does not support MTHFD2 being phosphorylated by ATM or DNA‐PK [37].

### 3.4 MTHFD2 is required for end resection following IR

**Fig. 5:**
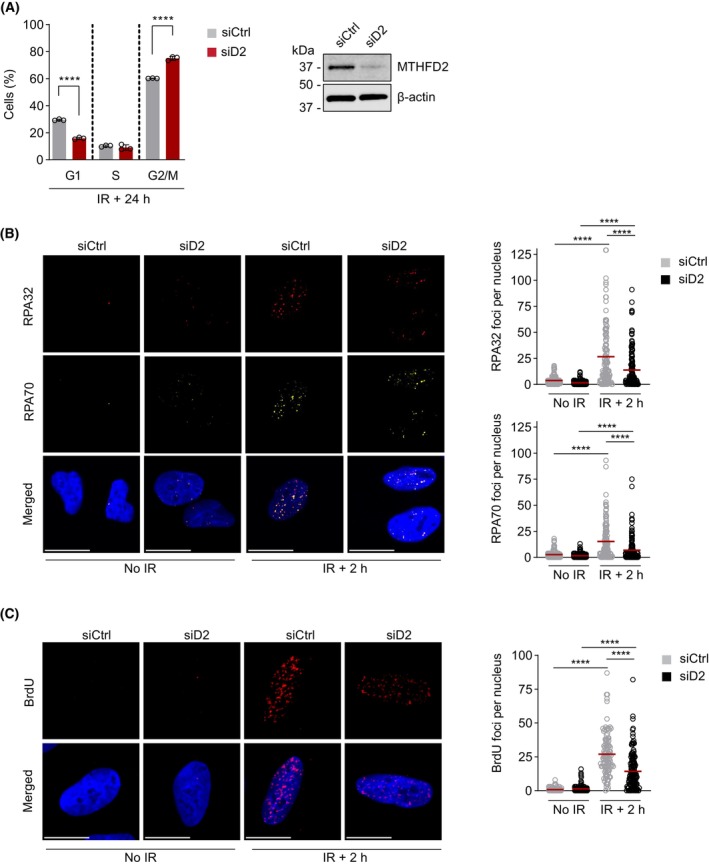
MTHFD2 is required for end resection following IR. (A) Cell cycle analysis upon *MTHFD2* depletion (10 nm, 24 h) and irradiation (IR) at 2 Gy in U2OS cells, analyzed 24 h post‐IR. A fraction of the samples was set aside for confirmation of knockdown by western blot. Bar graphs show the percentage of cells in each cell cycle phase as means ± SD, *n* = 3 independent experiments. ****P < 0.0001; one‐way ANOVA analysis with Tukey's multiple comparisons test. (B) Confocal analysis of RPA32 and RPA70 levels in U2OS cells following 24 h siRNA depletion of *MTHFD2* (siD2, 10 nm) and 2 h post‐IR (5 Gy). Shown is one out of three independent experiments. Scale bar on representative images, 10 μm. To the right, quantification of irradiation‐induced RPA32 and RPA70 foci per nucleus. Red bars indicate the mean, n (nuclei) = 161 (siCtrl, no IR), 153 (siD2 no IR), 123 (siCtrl, IR + 2 h) and 150 (siD2, IR + 2 h). ****P < 0.0001; one‐way ANOVA with Tukey's multiple comparisons test. (C) Confocal analysis of single‐stranded DNA (ssDNA) levels assessed using BrdU staining in U2OS cells treated with siCtrl or siMTHFD2 (siD2) for 48 h, labeled with 10 μm BrdU for 24 h, and subjected to irradiation (2 Gy, 2 h recovery) or left untreated. Shown is one out of three independent experiments. Scale bar on representative images, 10 μm. To the right, quantification of BrdU foci per nucleus. Red bars indicate the mean, n (nuclei) = 138 (siCtrl, no IR), 161 (siD2 no IR), 102 (siCtrl, IR + 2 h) and 139 (siD2, IR + 2 h). ****P < 0.0001; one‐way ANOVA with Tukey's multiple comparisons test.

In response to IR, ATM‐mediated DNA damage cell cycle checkpoints arrest cell cycle progression until the damage is repaired [4, 38]. Next, we sought to study the effects of *MTHFD2* knockdown on cell cycle progression upon IR. Interestingly, 24 h post‐IR we observed no change in S phase population of cells but a 20% increase in the G2/M population in *MTHFD2*‐depleted cells as compared to siCtrl cells (Fig. 5A). The G2/M arrest is particularly strong after IR and an increase in the G2/M population in *MTHFD2*‐depleted cells could indicate that there are remaining DSBs that keep cells arrested.

Next, we sought to dissect the steps of the DDR signaling cascade at which MTHFD2 comes into play. As nuclear MTHFD2 levels increase early following IR, we investigated early steps in the repair of IR‐induced DSBs, which involve binding and processing of DNA ends. The DNA end resection at the dsDNA end results in the generation of ssDNA, which is protected by the heterotrimeric Replication Protein A (RPA), consisting of the subunits RPA70, RPA32 and RPA14 [39]. Thus, we assessed whether silencing of *MTHFD2* affects end resection by staining for RPA70 and RPA32 foci following IR treatment. As expected, the treatment alone resulted in formation of both RPA70 and RPA32 foci indicating resection of the DNA ends to generate ssDNA (Fig. 5B). However, in U2OS cells transfected with siRNA targeting *MTHFD2* we failed to induce focal accumulation of either RPA70 and RPA32 foci as compared to control‐transfected cells (Fig. 5B). Resection at dsDNA ends and RPA foci formation primarily occur in the S and G2 phases of the cell cycle. As *MTHFD2* depletion increased the number of cells in the G2/M phases as determined by FACS analysis (Fig. 5A), the drop of RPA foci formation is not a result of altered cell cycle distribution following *MTHFD2* depletion.

We also wanted to use an alternative method to study end resection. The BrdU visualization method is commonly used to determine end resection and formation of ssDNA following IR and exploits the fact that only BrdU in ssDNA is identified and visualized in immunofluorescence [40]. Here, we observed significantly less BrdU foci upon irradiation in *MTHFD2*‐depleted cells, indicating diminished generation of ssDNA and resection (Fig. 5C). As MTHFD2 is recruited to sites of DNA damage it could have a direct role in resection. However, we found no evidence of MTHFD2 binding RPA70 directly by using co‐IP (Fig. S4B). Altogether, our data suggest that MTHFD2 is required for DNA end resection following IR.

### 3.5 MTHFD2 protein is required for efficient homologous recombination

**Fig. 6:**
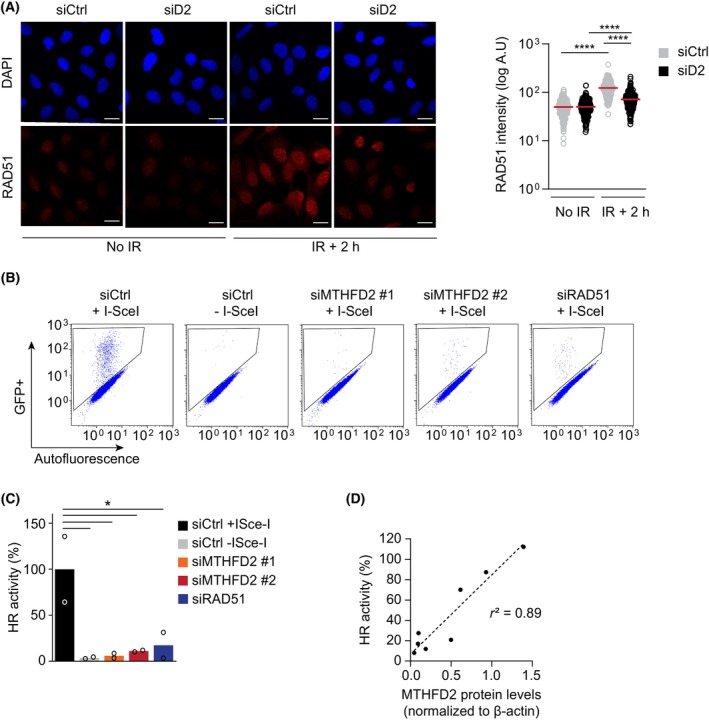
MTHFD2 protein is required for efficient homologous recombination. (A) Confocal analysis of nuclear RAD51 levels in U2OS cells following 24 h siRNA depletion of *MTHFD2* (siD2, 10 nm) and 2 h post‐irradiation (IR, 5 Gy). Shown is one out of three independent experiments. Scale bar on representative images, 20 μm. Red bars represent the mean intensity per nucleus, n (nuclei) = 309 (siCtrl, no IR), 203 (siD2 no IR), 216 (siCtrl, IR + 2 h) and 239 (siD2, IR + 2 h). ****P < 0.0001; one‐way ANOVA analysis with Šídák's multiple comparisons test. (B) DR‐GFP assay after 72 h treatment with indicated siRNA oligos in U2OS cells. Non‐targeting control (siCtrl) samples with and without I‐SceI nuclease co‐transfection were used as technical positive and negative controls, respectively. siRAD51 samples were used as a biological negative control. Representative dot plots with gated GFP‐positive cells are shown for one out of three experiments. (C) Homologous recombination (HR) activity after 72 h treatment of U2OS DR‐GFP cells with indicated siRNAs as assessed by flow cytometry analysis of GFP‐positive cell populations, related to (B). HR activity is displayed as the percentage of GFP‐positive cells relative to siCtrl + ISce‐I samples (set to 100% activity). Data are displayed as means, *n* = 2. Shown is one out of three independent experiments performed in duplicate. *P < 0.05; one‐way ANOVA analysis with Šídák's multiple comparisons test. siMTHFD2 #1 and #2 refer to two different siRNA oligos used. (D) Western blot protein quantification corresponding to U2OS DR‐GFP samples intended for flow cytometry analysis. MTHFD2 protein levels were correlated to each sample's HR activity. Pearson correlation coefficient of determination (*r* ^2^) is displayed for a linear regression model, *n* = 2 independent experiments.

During error‐free HR repair of DSBs, ssDNA overhangs at DNA serve as the handle to initiate the search for a homologous partner. The homology search and strand invasion is performed by the protein RAD51 that coats and replaces RPA on ssDNA overhangs [8]. Failure to carry out HR is often characterized by impairment of RAD51 foci formation [41]. Previously, we performed a genome‐wide RNAi screen (also using RAD51 foci formation as a readout) and identified MTHFD2 as a potential HR factor [42]. In line with these findings, the knockdown of *MTHFD2* also led to decreased IR‐induced nuclear accumulation of RAD51 compared to control‐transfected cells (Fig. 6A).

We further investigated the direct effect of MTHFD2 on HR repair activity using the DR‐GFP reporter assay that has been established in U2OS cells [43]. Co‐transfecting the cells with the I‐SceI nuclease expression vector introduces a DSBs specifically in one of the non‐functional GFP copies, which then in turn trigger a HR‐mediated gene conversion of the donor GFP sequence to restore a functional GFP sequence in about 5% of cells, as visualized by FACS analysis (Fig. 6B). Depletion of *MTHFD2* using two different siRNA oligonucleotide sequences reduced HR‐mediated gene conversion to a functional GFP protein to less than 20% compared to control‐transfected samples, affecting HR activity to a similar extent as the silencing of RAD51 [8] (Fig. 6B,C). In a similar DR‐GFP assay experiment, a fraction of the samples destined for flow cytometry analysis was collected for protein quantification. We observed that in the samples where the I‐SceI nuclease expression vector had been co‐transfected, HR proficiency significantly correlated with MTHFD2 protein levels (Fig. 6D). We also confirmed that transfection with I‐SceI nuclease did not affect MTHFD2 levels on its own. Therefore, we conclude that the presence of MTHFD2 is required for HR activity. Taken together, these data indicate that MTHFD2 supports HR repair by enabling the recruitment of the key HR factor RAD51.

The HR defect in *MTHFD2* siRNA‐depleted cells is unsurprising as it is involved in the early resection at DNA ends (Fig. 5), and failure to resect leads to impaired HR. The BRCA1 protein has an early and late role in DSB repair, and its early role is required for proper resection [44]. In response to DNA damage, BRCA1 becomes rapidly hyperphosphorylated by several kinases, including ATM, to allow efficient end resection and HR [45]. Here, we found that IR‐induced phosphorylation of BRCA1 at site S1524 was impaired in siMTHFD2 and *MTHFD2* ^−/−^ cells, indicating that MTHFD2 affects early steps in DSB repair (Fig. S5A,B). Interestingly, we did not observe any difference in total RPA70 and RAD51 levels in *MTHFD2*‐depleted cells, demonstrating that the failure of forming foci is owing to failure of recruitment of RPA and RAD51 and not due to decreased protein levels (Fig. S5A).

In addition to promoting HR, BRCA1 protein is also involved in non‐homologous end joining as part of early response [46, 47]. Here, we could also observe a defect in NHEJ following depletion of *MTHFD2* (Fig. S5C), in line with earlier findings that suggest the involvement of MTHFD2 in NHEJ as well [26].

### 3.6 Loss of *MTHFD2* impairs IR‐induced DSB repair

**Fig. 7:**
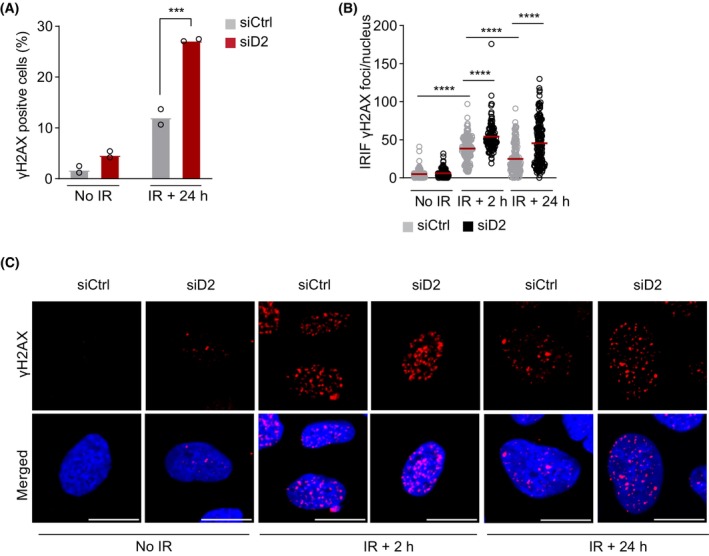
Loss of *MTHFD2* impairs irradiation‐induced double‐strand break (DSB) repair. (A) Flow cytometry analysis of γH2AX levels upon *MTHFD2* depletion (10 nm, 24 h) and ionizing radiation (IR) at 2 Gy in U2OS cells, analyzed 24 h post‐IR. Bar graphs show the percentage of γH2AX‐positive cells, as means ± SD, *n* = 2 independent experiments. ***P < 0.001, one‐way ANOVA analysis with Tukey's multiple comparisons test. (B) Confocal analysis of DNA damage levels assessed using ɣH2AX staining in U2OS cells treated with siCtrl or siMTHFD2 (siD2) for 24 h and subjected to irradiation (5 Gy, 2 and 24 h recovery), or left untreated. Shown is a quantification of irradiation‐induced ɣH2AX foci per nucleus for one out of three independent experiments. Red bars indicate the mean, n (nuclei) = 131 (siCtrl, no IR), 151 (siD2 no IR), 157 (siCtrl, IR + 2 h), 115 (siD2, IR + 2 h), 134 (siCtrl, IR + 24 h) and 174 (siD2, IR + 24 h). ****P < 0.0001; one‐way ANOVA with Šídák's multiple comparisons test. (C) Representative images related to (B). Shown is a representative experiment of *n* = 3 independent experiments. Scale bar on representative images, 10 μm.

Given the observed HR defect upon *MTHFD2* knockdown, we sought to study the functional consequences of defective HR upon IR in *MTHFD2*‐depleted cells. Consistent with a larger proportion of cells arrested in the G2/M phase (Fig. 5A), *MTHFD2*‐depleted cells also displayed increased levels of γH2AX 24 h post‐IR, suggesting delayed or impaired repair of IR‐induced DNA lesions (Fig. 7). In summary, loss of MTHFD2 blocks HR repair of IR‐induced DSBs by preventing RPA and RAD51 recruitment, resulting in high levels of residual DNA damage as assessed by γH2AX and prolonged cell cycle arrest.

## 4 Discussion

While the mitochondrial role of MTHFD2 to promote rapid growth is well established [48], the nuclear role of MTHFD2 is more elusive. One report ascribed MTHFD2 to have a specific non‐catalytic role in p53‐defective cells through regulation of PARP3‐dependent end joining [26]. Others report that MTHFD2 binds CDK1 and EXO1 and promotes repair in mouse pluripotent stem cells [27]. The data presented in this report support an early role of MTHFD2 in the DNA damage response following IR treatment, already at the resection stage, which we show is mediated through the ATM and DNA‐PK kinases. We also demonstrate impairment of BRCA1 phosphorylation, altogether supporting MTHFD2 having a role early in DSB repair and affecting both HR and NHEJ. Our observation that MTHFD2 affects resection is supported by *MTHFD2*‐depleted cells showing reduced IR‐induced RPA foci formation and visualization of ssDNA with BrdU. Hence, our data support earlier work [26, 27] suggesting a function for MTHFD2 in the early steps of DSB repair. The role of MTHFD2 in DNA repair may have translational implications. Here, we demonstrate that especially nuclear MTHFD2 protein levels increase upon IR and DNA damage also enhances association of MTHFD2 to chromatin. Depleting *MTHFD2* abolishes IR‐induced recruitment of RPA and RAD51, blocks HR activity and ultimately results in a high amount of unresolved DSBs and accumulation in the G2/M phase. MTHFD2 is described as the second (among > 20 000) most overexpressed gene in cancer versus non‐malignant tissue, thus being highly cancer‐specific [20]. Therefore, inhibiting MTHFD2 may specifically affect and impair DSB repair in cancer cells versus normal. Our data provide a rationale for future studies focusing on MTHFD1/2 inhibitors [23] and whether they may selectively sensitize cancer cells during radiotherapy.

## 5 Conclusions

In summary, here we present evidence that the MTHFD2 protein is recruited, by ATM and DNA‐PK, to the nucleus upon IR treatment and that MTHFD2 promotes DNA end resection, RPA and RAD51 loading and HR repair. We propose that targeting MTHFD2 provides a potentially clinically relevant opportunity to selectively introduce HR deficiency in cancer cells, thereby sensitizing repair‐proficient tumors to IR.

## Conflict of interest

One‐carbon therapeutics AB (OCT) develops MTHFD1/2 inhibitors commercially and AS is employed by OCT. PM, NB, CC, EW, AR, UWB, OM, AS and TH have direct or indirect financial interests in OCT.

## Author contributions

Conceptualization: NB, PM, OM, UWB and TH; Methodology, Investigation and Formal Analysis: PM, NB, CC, HS, KS, JS, CMF, AH, JA, EW, AR, OM, TH and UWB; Resources: TH; Writing – Original Draft: NB, OM, TH; Writing – Review and Editing: PM, AS, and TH; Visualization: PM, NB; Supervision: PM, NB, AS, OM, UWB and TH; Project Administration: PM, NB, AS, UWB and TH; Funding Acquisition: TH.

## Supporting information


**Fig. S1.** MTHFD2 but not MTHFD1 accumulates in the nucleus following IR treatment.
**Fig. S2.** *MTHFD2* silencing impairs cancer cell survival after irradiation.
**Fig. S3.** Depletion of *MTHFD2* hampers cell proliferation following irradiation.
**Fig. S4.** MTHFD2 does not interact with ATM, DNA‐PK or RPA70.
**Fig. S5.** MTHFD2 promotes DSB repair.
**Fig. S6.** Uncropped immunoblots for Fig. 1A.
**Fig. S7.** Uncropped immunoblots for Fig. 1C.
**Fig. S8.** Uncropped immunoblots for Fig. 2C.
**Fig. S9.** Uncropped immunoblots for Figs 3E and 5A.
**Fig. S10.** Uncropped immunoblots for Fig. S2B.
**Fig. S11.** Uncropped immunoblots for Fig. S2D.
**Fig. S12.** Uncropped immunoblots for Fig. S4A,B.
**Fig. S13.** Uncropped immunoblots for Fig. S5A.
**Fig. S14.** Uncropped immunoblots for Fig. S5B.
**Fig. S15.** Raw image data of representative wells from the clonogenic survival assay performed in U2OS cells.
**Fig. S16.** Raw image data of representative wells from the clonogenic survival assay performed in HCT116 cells.

## Data Availability

All data generated or analyzed during this study, including source data, can be found in the article or in the Supporting Information. Uncropped immunoblots are shown in Figs S6–S14 and raw image data on representative colonies is shown in Figs S15 and S16. Additional data generated during this study and relevant information are available from the corresponding authors upon reasonable request.
