# Supplementary material for: The one‐carbon metabolic enzyme MTHFD2 promotes resection and homologous recombination after ionizing radiation
Source: Mol Oncol. 2024 Mar 27;18(9):2179–95. doi: 10.1002/1878-0261.13645 (PMC11467796; doi:10.1002/1878-0261.13645)
Supplement: Supplementary file 1 — Fig. S1. MTHFD2 but not MTHFD1 accumulates in the nucleus following IR treatment. Fig. S2. MTHFD2 silencing impairs cancer cell survival after irradiation. Fig. S3. Depletion of MTHFD2 hampers cell proliferation following irradiation. Fig. S4. MTHFD2 does not interact with ATM, DNA‐PK or RPA70. Fig. S5. MTHFD2 promotes DSB repair. Fig. S6. Uncropped immunoblots for Fig. 1A. Fig. S7. Uncropped immunoblots for Fig. 1C. Fig. S8. Uncropped immunoblots for Fig. 2C. Fig. S9. Uncropped immunoblots for Figs 3E and 5A. Fig. S10. Uncropped immunoblots for Fig. S2B. Fig. S11. Uncropped immunoblots for Fig. S2D. Fig. S12. Uncropped immunoblots for Fig. S4A,B. Fig. S13. Uncropped immunoblots for Fig. S5A. Fig. S14. Uncropped immunoblots for Fig. S5B. Fig. S15. Raw image data of representative wells from the clonogenic survival assay performed in U2OS cells. Fig. S16. Raw image data of representative wells from the clonogenic survival assay performed in HCT116 cells. [file MOL2-18-2179-s001.zip › mol213645-sup-0017-Supinfo.docx]

**Supplementary Information**

**The one-carbon metabolic enzyme MTHFD2 promotes resection and homologous recombination after ionizing radiation**

Marttila *et al.*

**SUPPLEMENTARY FIGURE LEGENDS**

**Supplementary Figure 1.** **MTHFD2 but not MTHFD1 accumulates in the nucleus following IR treatment.**

(a) Confocal analysis of co-localization between MTHFD2 and γH2AX, in U2OS cells subjected to irradiation (5 Gy, 2h recovery), or left untreated. Shown is a representative of n = 3 independent experiments. Scale bar on representative images, 10 μm

(b) Correlation plots of the nuclear intensities of MTHFD2 and ɣH2AX shown in panel (a) as quantified using CellProfiler, n (nuclei) = 186 (no IR) and 152 (IR); Pearson correlation test.

(c) Validations of MTHFD2 targeting antibodies in SW620 wildtype and SW620 *MTHFD2^-/-^* cells. The Abcam antibody (ab56772) was used in the immunofluorescence experiments described in this study.

(d) Confocal analysis of MTHFD1 in U2OS cells following treatment with siCtrl or siMTHFD1 (siD1) at 10 nM for 24 h. Shown is a representative experiment out of two independent experiments. Scale bar, 10 μm. Bottom, a scatter dot plot showing nuclear MTHFD1 intensity as quantified using CellProfiler. Red bars indicate the mean, n (nuclei) = 214 (siCtrl) and 152 (siD1) 64. ****P<0.0001; unpaired t-test.

(e) Confocal analysis of subcellular localization of MTHFD1 and induction γH2AX in U2OS cells subjected to irradiation (5 Gy) and fixed at indicated time points post-irradiation. Shown is one out of two independent experiments. Scale bar on representative images, 10 μm. To the right, scatter dot plots showing nuclear MTHFD1 and ɣH2AX intensities. Red bars represent mean intensities, n (nuclei) = 174 (no IR), 197 (1h post-IR), 230 (2h post-IR) and 228 (4h post-IR). *** P<0.001, **** P<0.0001; one-way ANOVA analysis with Dunnett’s multiple comparisons test.

**Supplementary Figure 2. *MTHFD2* silencing impairs cancer cell survival after irradiation.**

(a) Representative images of a clonogenic assay in U2OS cells as quantified in Fig. 3a, n = 2 independent experiments.

(b) Western blots depict validation of the siRNA knockdown efficiency in U2OS cells irradiated at indicated doses and harvested 4h post-IR. α-Tubulin was used as a loading control. Related to Fig. 3a and Supplementary Fig. 2a. Shown is a representative experiment of n = 2 independent experiments.

(c) Representative images of a clonogenic assay in HCT116 cells as quantified in Fig. 3b, n = 2 independent experiments.

(d) Western blots depict validation of the siRNA knockdown efficiency in HCT116 cells irradiated at indicated doses and harvested 4h post-IR. Histone H3 was used as a loading control. Related to Fig. 3b and Supplementary Fig. 2c. Shown is a representative experiment of n = 2 independent experiments.

**Supplementary Figure 3. Depletion of *MTHFD2* hampers cell proliferation following irradiation.**

Representative images of fields used to calculate confluency of U2OS and HCT116 cells as reported in Fig. 3c,d, n = 3 independent experiments. Images were acquired with Tecan Spark Cyto plate reader (brightfield microscope, 4x magnification).

**Supplementary Figure 4. MTHFD2 does not interact with ATM, DNA-PK or RPA70.**

(a) Co-immunoprecipitation of endogenous MTHFD2, ATM and DNA-PKcs with mouse (Ms) anti-MTHFD2 primary antibody. U2OS cells were irradiated at 5 Gy (2h recovery) or left untreated before harvesting, followed by immunoprecipitation and Western blot analysis. Shown is a representative experiment of n = 2.

(b) Co-immunoprecipitation of endogenous MTHFD2 and RPA70 with mouse (Ms) anti-MTHFD2 primary antibody. U2OS cells were irradiated at 5 Gy (2h recovery) or left untreated before harvesting, followed by immunoprecipitation and Western blot analysis. Shown is a representative experiment of n = 2.

**Supplementary Figure 5. MTHFD2 promotes DSB repair.**

(a) U2OS cells were treated with siRNAs targeting *MTHFD1* (siD1) or *MTHFD2* (siD2) or non-targeting control (siCtrl) for 24h, subjected to irradiation (2 Gy) and harvested 4h after IR. Whole cell extracts were prepared and protein levels were analyzed using Western blot with indicated antibodies. α-Tubulin was used as a loading control. Shown is a representative experiment of n = 3.

(b) SW620 wildtype and SW620 *MTHFD2-/-* cells were subjected to irradiation at 5 Gy (2h recovery), followed by cell fractionations for isolation of cytosolic/mitochondrial (C/M) proteins and chromatin-bound (CB) proteins). Protein levels were analyzed using Western blot with indicated antibodies. α-Tubulin and Histone H3 were used as loading controls. Shown is a representative experiment of n = 2.

(c) NHEJ activity after 72h treatment of U2OS DR-GFP cells with indicated siRNAs or DNA-PKi (NU-7441, 2 µM) as assessed by flow cytometry analysis of GFP-positive cell populations. NHEJ activity is displayed as the percentage of GFP-positive cells relative to siCtrl + ISce-I samples (set to 100% activity). Data are displayed as means, n = 2. * P<0.05, **P < 0.01, ***P < 0.001; one-way ANOVA analysis with Dunnett’s multiple comparisons test.

**Supplementary Figure 6. Uncropped immunoblots for Figure 1a.**

Dotted lines indicate cropped area.

**Supplementary Figure 7. Uncropped immunoblots for Figure 1c.**

Dotted lines indicate cropped area.

**Supplementary Figure 8. Uncropped immunoblots for Figure 2c.**

Dotted lines indicate cropped area.

**Supplementary Figure 9. Uncropped immunoblots for Figures 3e and 5a.**

(a) Unprocessed images of blots related to Figure 3e.

(b) Unprocessed images of blots related to Figure 5a. Dotted lines indicate cropped area.

**Supplementary Figure 10. Uncropped immunoblots for Supplementary Figure 2b.**

Dotted lines indicate cropped area.

**Supplementary Figure 11. Uncropped immunoblots for Supplementary Figure 2d.**

Dotted lines indicate cropped area.

**Supplementary Figure 12. Uncropped immunoblots for Supplementary Figure 4a, b.**

(a) Unprocessed images of blots related to Supplementary Figure 4a.

(b) Unprocessed images of blots related to Supplementary Figure 4b. Dotted lines indicate cropped area.

**Supplementary Figure 13. Uncropped immunoblots for Supplementary Figure 5a.**

Dotted lines indicate cropped area.

**Supplementary Figure 14. Uncropped immunoblots for Supplementary Figure 5b.**

Dotted lines indicate cropped area.

**Supplementary Figure 15. Raw image data of representative wells from the clonogenic survival assay performed in U2OS cells.** Unprocessed images related to Supplementary Figure 2a. Dotted lines indicate cropped area.

**Supplementary Figure 16. Raw image data of representative wells from the clonogenic survival assay performed in HCT116 cells.** Unprocessed images related to Supplementary Figure 2c. Dotted lines indicate cropped area.
