# Supplementary figures and images for: The one‐carbon metabolic enzyme MTHFD2 promotes resection and homologous recombination after ionizing radiation
Source: Mol Oncol. 2024 Mar 27;18(9):2179–95. doi: 10.1002/1878-0261.13645 (PMC11467796; doi:10.1002/1878-0261.13645)

Supplementary Figure 1.

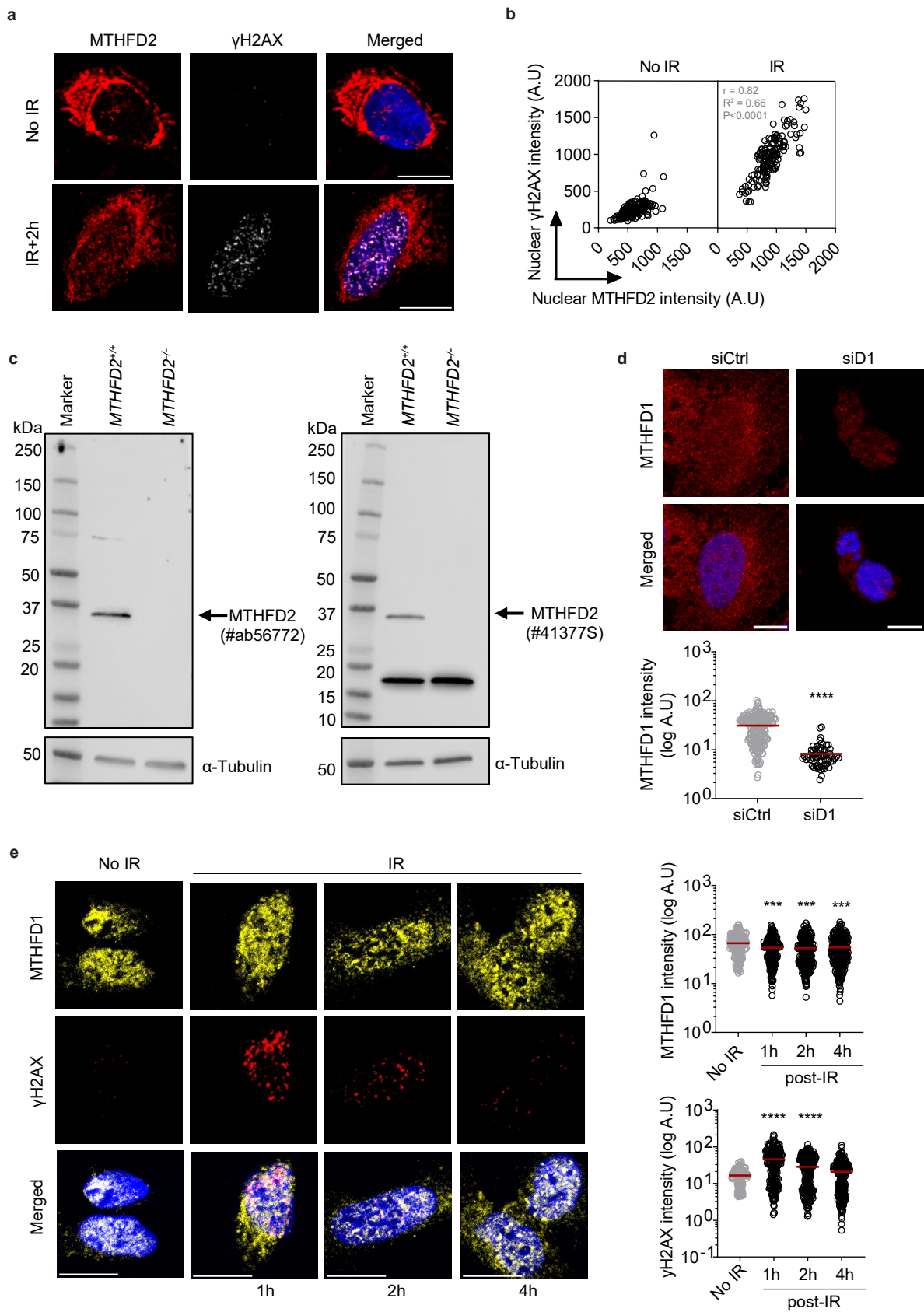

Supplement: Supplementary file 1 — Fig. S1. MTHFD2 but not MTHFD1 accumulates in the nucleus following IR treatment. Fig. S2. MTHFD2 silencing impairs cancer cell survival after irradiation. Fig. S3. Depletion of MTHFD2 hampers cell proliferation following irradiation. Fig. S4. MTHFD2 does not interact with ATM, DNA‐PK or RPA70. Fig. S5. MTHFD2 promotes DSB repair. Fig. S6. Uncropped immunoblots for Fig. 1A. Fig. S7. Uncropped immunoblots for Fig. 1C. Fig. S8. Uncropped immunoblots for Fig. 2C. Fig. S9. Uncropped immunoblots for Figs 3E and 5A. Fig. S10. Uncropped immunoblots for Fig. S2B. Fig. S11. Uncropped immunoblots for Fig. S2D. Fig. S12. Uncropped immunoblots for Fig. S4A,B. Fig. S13. Uncropped immunoblots for Fig. S5A. Fig. S14. Uncropped immunoblots for Fig. S5B. Fig. S15. Raw image data of representative wells from the clonogenic survival assay performed in U2OS cells. Fig. S16. Raw image data of representative wells from the clonogenic survival assay performed in HCT116 cells. [file MOL2-18-2179-s001.zip › mol213645-sup-0001-FigureS1.pdf]

**Supplementary Figure 2.**

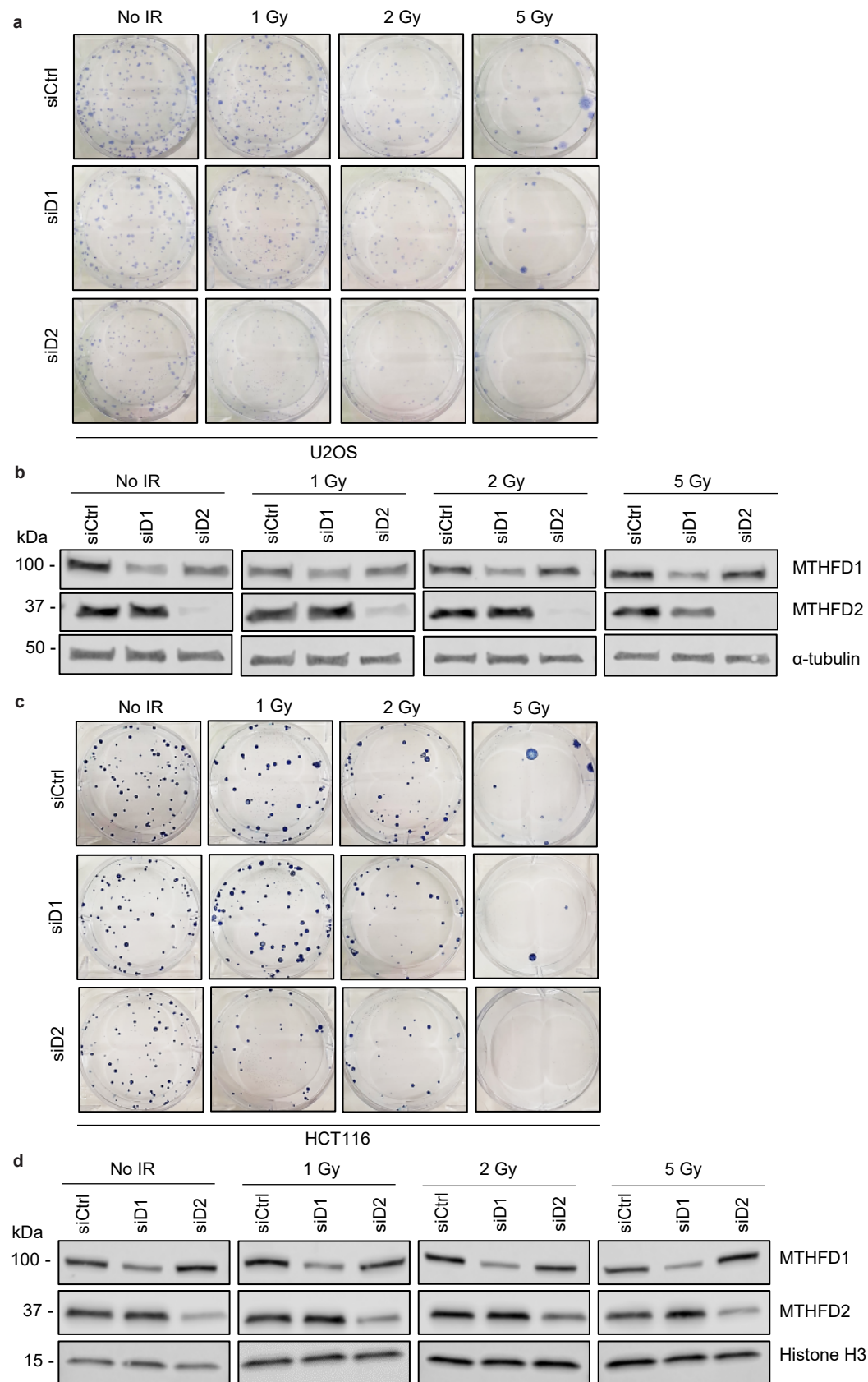

Supplement: Supplementary file 1 — Fig. S1. MTHFD2 but not MTHFD1 accumulates in the nucleus following IR treatment. Fig. S2. MTHFD2 silencing impairs cancer cell survival after irradiation. Fig. S3. Depletion of MTHFD2 hampers cell proliferation following irradiation. Fig. S4. MTHFD2 does not interact with ATM, DNA‐PK or RPA70. Fig. S5. MTHFD2 promotes DSB repair. Fig. S6. Uncropped immunoblots for Fig. 1A. Fig. S7. Uncropped immunoblots for Fig. 1C. Fig. S8. Uncropped immunoblots for Fig. 2C. Fig. S9. Uncropped immunoblots for Figs 3E and 5A. Fig. S10. Uncropped immunoblots for Fig. S2B. Fig. S11. Uncropped immunoblots for Fig. S2D. Fig. S12. Uncropped immunoblots for Fig. S4A,B. Fig. S13. Uncropped immunoblots for Fig. S5A. Fig. S14. Uncropped immunoblots for Fig. S5B. Fig. S15. Raw image data of representative wells from the clonogenic survival assay performed in U2OS cells. Fig. S16. Raw image data of representative wells from the clonogenic survival assay performed in HCT116 cells. [file MOL2-18-2179-s001.zip › mol213645-sup-0002-FigureS2.pdf]

**Supplementary Figure 3.**

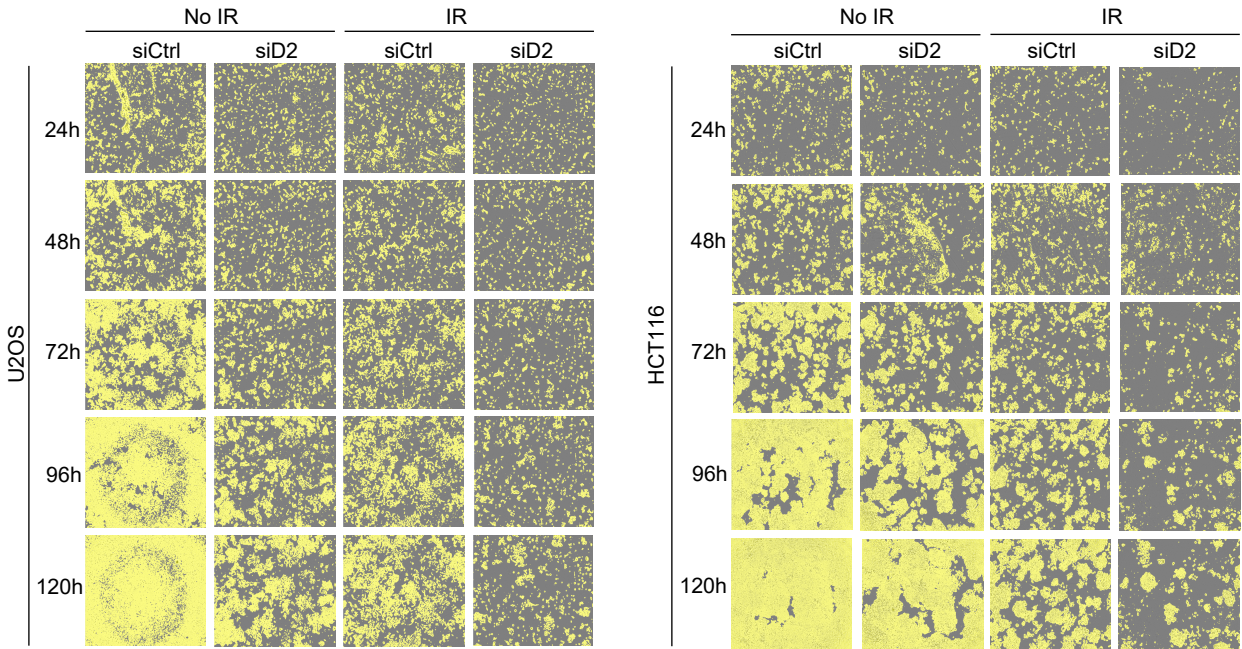

Supplement: Supplementary file 1 — Fig. S1. MTHFD2 but not MTHFD1 accumulates in the nucleus following IR treatment. Fig. S2. MTHFD2 silencing impairs cancer cell survival after irradiation. Fig. S3. Depletion of MTHFD2 hampers cell proliferation following irradiation. Fig. S4. MTHFD2 does not interact with ATM, DNA‐PK or RPA70. Fig. S5. MTHFD2 promotes DSB repair. Fig. S6. Uncropped immunoblots for Fig. 1A. Fig. S7. Uncropped immunoblots for Fig. 1C. Fig. S8. Uncropped immunoblots for Fig. 2C. Fig. S9. Uncropped immunoblots for Figs 3E and 5A. Fig. S10. Uncropped immunoblots for Fig. S2B. Fig. S11. Uncropped immunoblots for Fig. S2D. Fig. S12. Uncropped immunoblots for Fig. S4A,B. Fig. S13. Uncropped immunoblots for Fig. S5A. Fig. S14. Uncropped immunoblots for Fig. S5B. Fig. S15. Raw image data of representative wells from the clonogenic survival assay performed in U2OS cells. Fig. S16. Raw image data of representative wells from the clonogenic survival assay performed in HCT116 cells. [file MOL2-18-2179-s001.zip › mol213645-sup-0003-FigureS3.pdf]

Supplementary Figure 4.

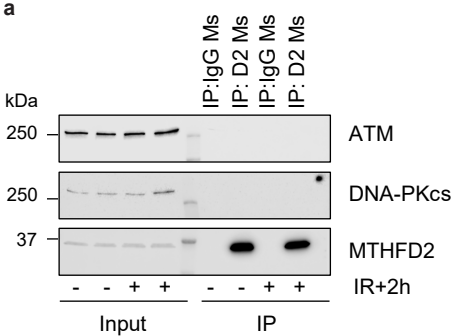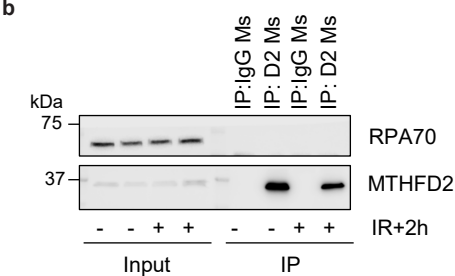

Supplement: Supplementary file 1 — Fig. S1. MTHFD2 but not MTHFD1 accumulates in the nucleus following IR treatment. Fig. S2. MTHFD2 silencing impairs cancer cell survival after irradiation. Fig. S3. Depletion of MTHFD2 hampers cell proliferation following irradiation. Fig. S4. MTHFD2 does not interact with ATM, DNA‐PK or RPA70. Fig. S5. MTHFD2 promotes DSB repair. Fig. S6. Uncropped immunoblots for Fig. 1A. Fig. S7. Uncropped immunoblots for Fig. 1C. Fig. S8. Uncropped immunoblots for Fig. 2C. Fig. S9. Uncropped immunoblots for Figs 3E and 5A. Fig. S10. Uncropped immunoblots for Fig. S2B. Fig. S11. Uncropped immunoblots for Fig. S2D. Fig. S12. Uncropped immunoblots for Fig. S4A,B. Fig. S13. Uncropped immunoblots for Fig. S5A. Fig. S14. Uncropped immunoblots for Fig. S5B. Fig. S15. Raw image data of representative wells from the clonogenic survival assay performed in U2OS cells. Fig. S16. Raw image data of representative wells from the clonogenic survival assay performed in HCT116 cells. [file MOL2-18-2179-s001.zip › mol213645-sup-0004-FigureS4.pdf]

**Supplementary Figure 5.**

**a**

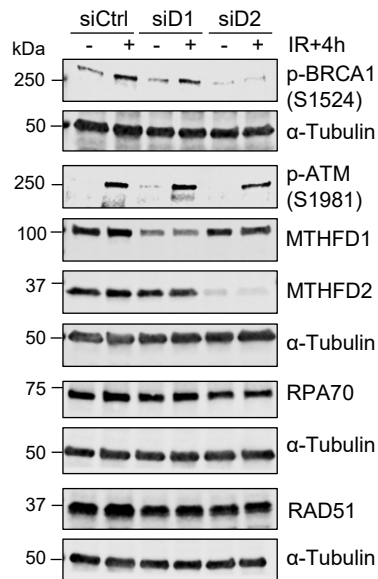

**b**

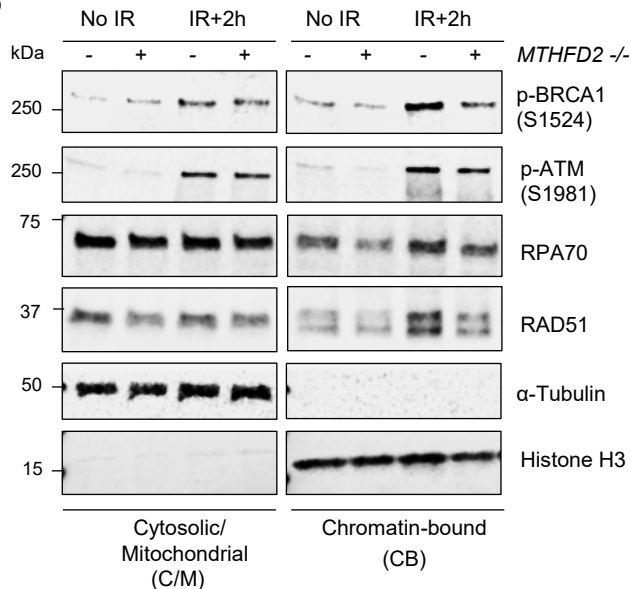

**c**

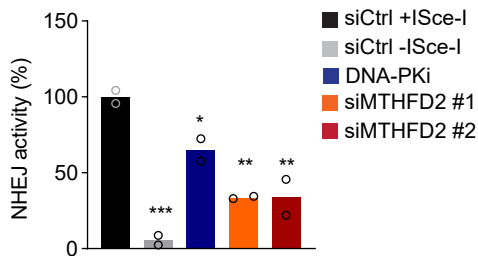

Supplement: Supplementary file 1 — Fig. S1. MTHFD2 but not MTHFD1 accumulates in the nucleus following IR treatment. Fig. S2. MTHFD2 silencing impairs cancer cell survival after irradiation. Fig. S3. Depletion of MTHFD2 hampers cell proliferation following irradiation. Fig. S4. MTHFD2 does not interact with ATM, DNA‐PK or RPA70. Fig. S5. MTHFD2 promotes DSB repair. Fig. S6. Uncropped immunoblots for Fig. 1A. Fig. S7. Uncropped immunoblots for Fig. 1C. Fig. S8. Uncropped immunoblots for Fig. 2C. Fig. S9. Uncropped immunoblots for Figs 3E and 5A. Fig. S10. Uncropped immunoblots for Fig. S2B. Fig. S11. Uncropped immunoblots for Fig. S2D. Fig. S12. Uncropped immunoblots for Fig. S4A,B. Fig. S13. Uncropped immunoblots for Fig. S5A. Fig. S14. Uncropped immunoblots for Fig. S5B. Fig. S15. Raw image data of representative wells from the clonogenic survival assay performed in U2OS cells. Fig. S16. Raw image data of representative wells from the clonogenic survival assay performed in HCT116 cells. [file MOL2-18-2179-s001.zip › mol213645-sup-0005-FigureS5.pdf]

**Supplementary Figure 6.**

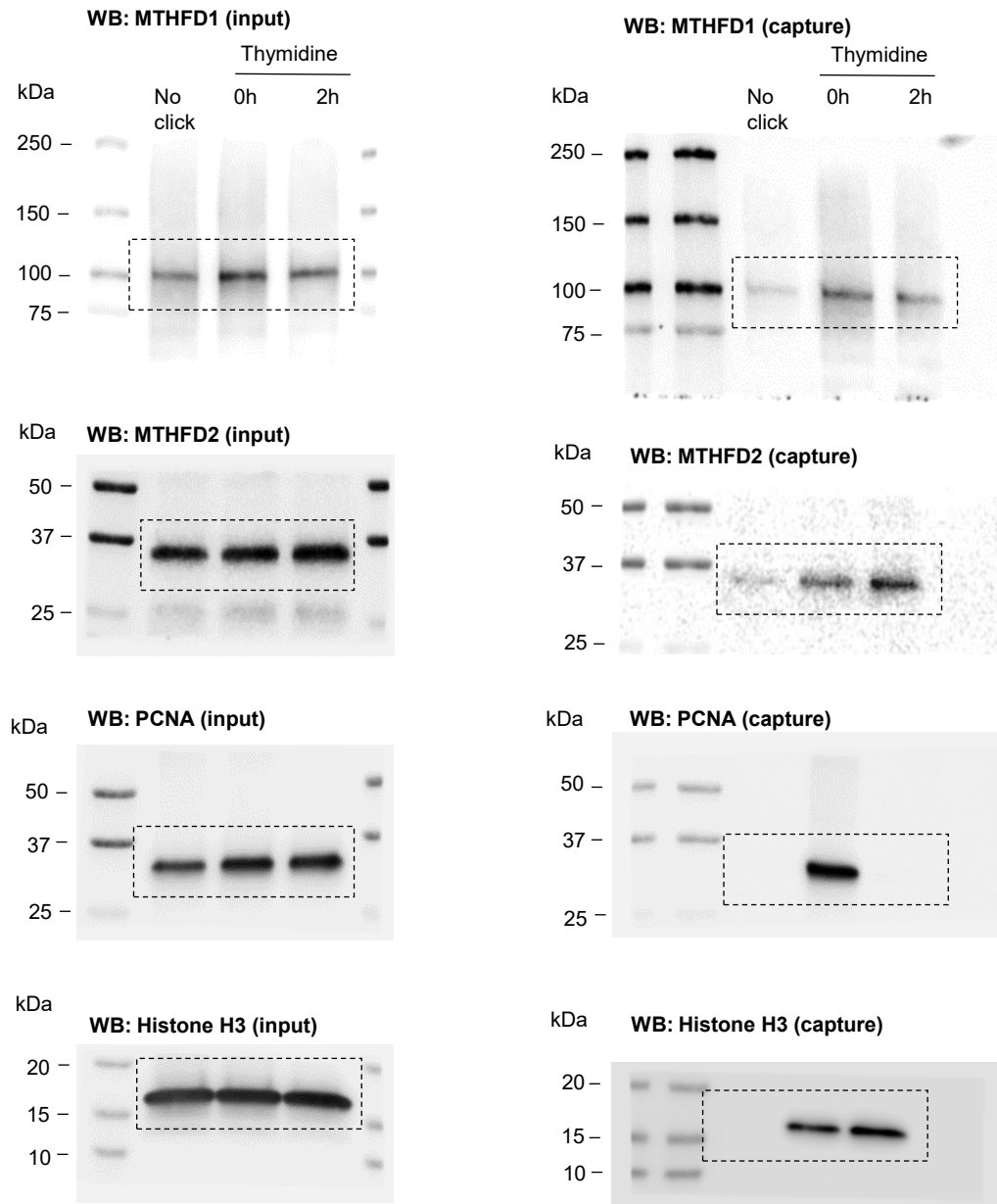

Supplement: Supplementary file 1 — Fig. S1. MTHFD2 but not MTHFD1 accumulates in the nucleus following IR treatment. Fig. S2. MTHFD2 silencing impairs cancer cell survival after irradiation. Fig. S3. Depletion of MTHFD2 hampers cell proliferation following irradiation. Fig. S4. MTHFD2 does not interact with ATM, DNA‐PK or RPA70. Fig. S5. MTHFD2 promotes DSB repair. Fig. S6. Uncropped immunoblots for Fig. 1A. Fig. S7. Uncropped immunoblots for Fig. 1C. Fig. S8. Uncropped immunoblots for Fig. 2C. Fig. S9. Uncropped immunoblots for Figs 3E and 5A. Fig. S10. Uncropped immunoblots for Fig. S2B. Fig. S11. Uncropped immunoblots for Fig. S2D. Fig. S12. Uncropped immunoblots for Fig. S4A,B. Fig. S13. Uncropped immunoblots for Fig. S5A. Fig. S14. Uncropped immunoblots for Fig. S5B. Fig. S15. Raw image data of representative wells from the clonogenic survival assay performed in U2OS cells. Fig. S16. Raw image data of representative wells from the clonogenic survival assay performed in HCT116 cells. [file MOL2-18-2179-s001.zip › mol213645-sup-0006-FigureS6.pdf]

Supplementary Figure 7.

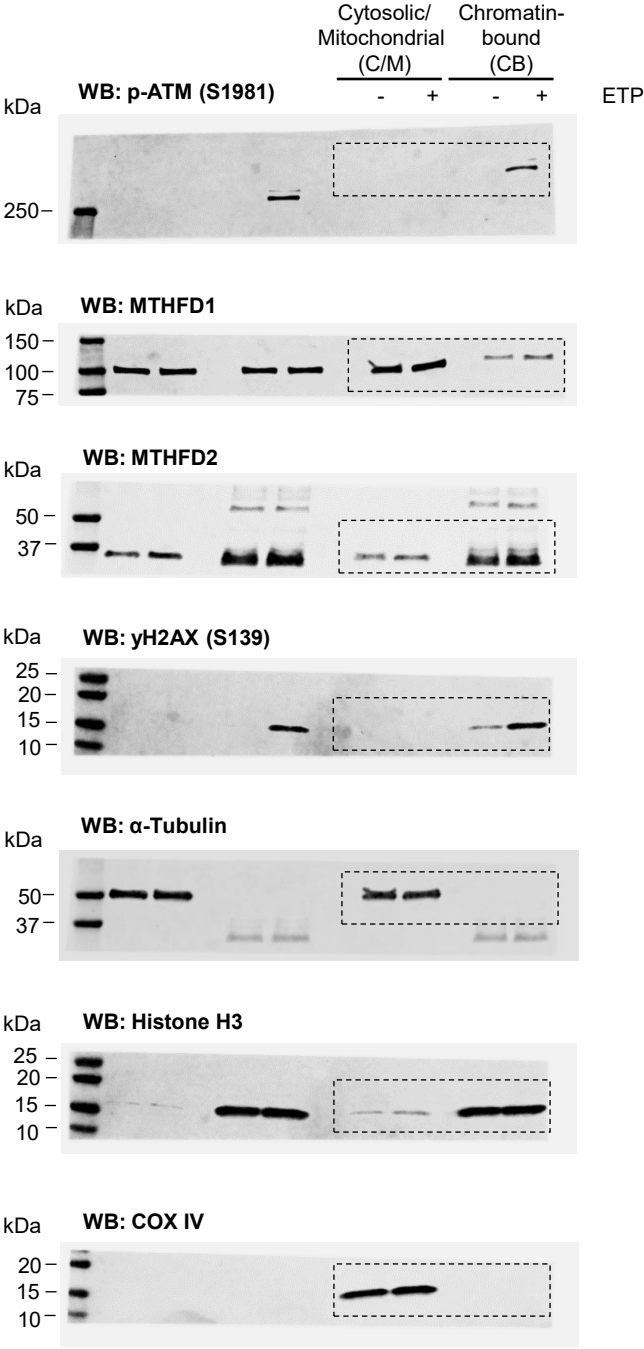

Supplement: Supplementary file 1 — Fig. S1. MTHFD2 but not MTHFD1 accumulates in the nucleus following IR treatment. Fig. S2. MTHFD2 silencing impairs cancer cell survival after irradiation. Fig. S3. Depletion of MTHFD2 hampers cell proliferation following irradiation. Fig. S4. MTHFD2 does not interact with ATM, DNA‐PK or RPA70. Fig. S5. MTHFD2 promotes DSB repair. Fig. S6. Uncropped immunoblots for Fig. 1A. Fig. S7. Uncropped immunoblots for Fig. 1C. Fig. S8. Uncropped immunoblots for Fig. 2C. Fig. S9. Uncropped immunoblots for Figs 3E and 5A. Fig. S10. Uncropped immunoblots for Fig. S2B. Fig. S11. Uncropped immunoblots for Fig. S2D. Fig. S12. Uncropped immunoblots for Fig. S4A,B. Fig. S13. Uncropped immunoblots for Fig. S5A. Fig. S14. Uncropped immunoblots for Fig. S5B. Fig. S15. Raw image data of representative wells from the clonogenic survival assay performed in U2OS cells. Fig. S16. Raw image data of representative wells from the clonogenic survival assay performed in HCT116 cells. [file MOL2-18-2179-s001.zip › mol213645-sup-0007-FigureS7.pdf]

Supplementary Figure 8.

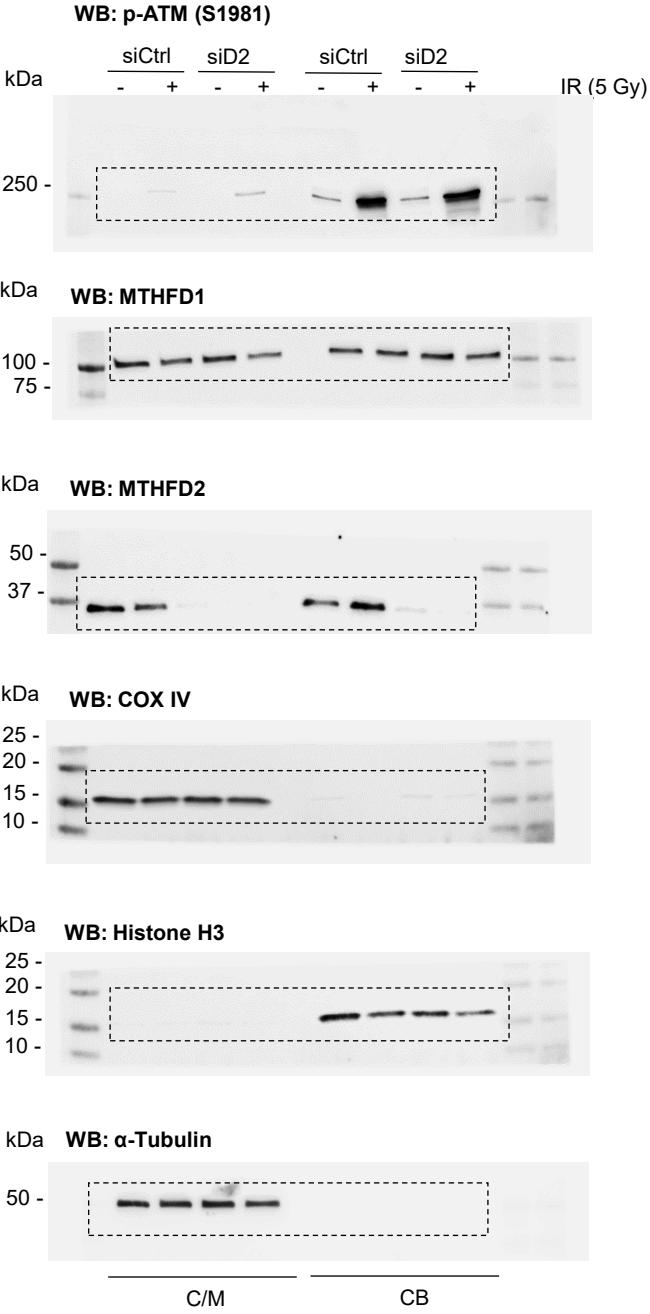

Supplement: Supplementary file 1 — Fig. S1. MTHFD2 but not MTHFD1 accumulates in the nucleus following IR treatment. Fig. S2. MTHFD2 silencing impairs cancer cell survival after irradiation. Fig. S3. Depletion of MTHFD2 hampers cell proliferation following irradiation. Fig. S4. MTHFD2 does not interact with ATM, DNA‐PK or RPA70. Fig. S5. MTHFD2 promotes DSB repair. Fig. S6. Uncropped immunoblots for Fig. 1A. Fig. S7. Uncropped immunoblots for Fig. 1C. Fig. S8. Uncropped immunoblots for Fig. 2C. Fig. S9. Uncropped immunoblots for Figs 3E and 5A. Fig. S10. Uncropped immunoblots for Fig. S2B. Fig. S11. Uncropped immunoblots for Fig. S2D. Fig. S12. Uncropped immunoblots for Fig. S4A,B. Fig. S13. Uncropped immunoblots for Fig. S5A. Fig. S14. Uncropped immunoblots for Fig. S5B. Fig. S15. Raw image data of representative wells from the clonogenic survival assay performed in U2OS cells. Fig. S16. Raw image data of representative wells from the clonogenic survival assay performed in HCT116 cells. [file MOL2-18-2179-s001.zip › mol213645-sup-0008-FigureS8.pdf]

Supplementary Figure 9.

a

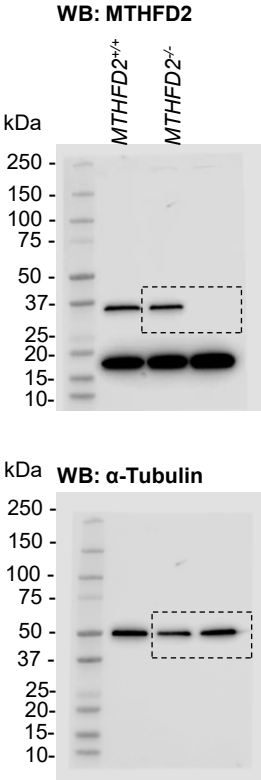

b

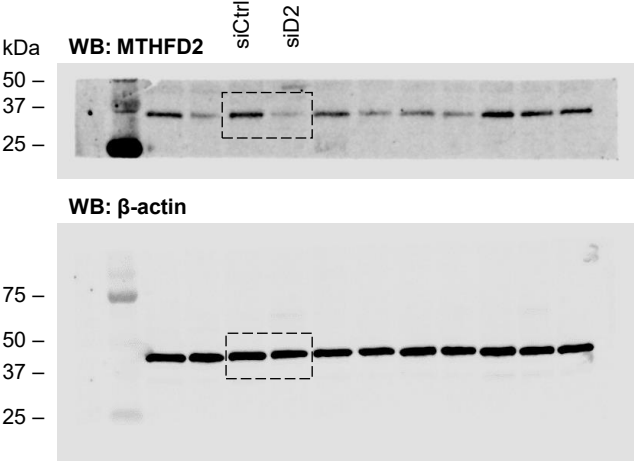

Supplement: Supplementary file 1 — Fig. S1. MTHFD2 but not MTHFD1 accumulates in the nucleus following IR treatment. Fig. S2. MTHFD2 silencing impairs cancer cell survival after irradiation. Fig. S3. Depletion of MTHFD2 hampers cell proliferation following irradiation. Fig. S4. MTHFD2 does not interact with ATM, DNA‐PK or RPA70. Fig. S5. MTHFD2 promotes DSB repair. Fig. S6. Uncropped immunoblots for Fig. 1A. Fig. S7. Uncropped immunoblots for Fig. 1C. Fig. S8. Uncropped immunoblots for Fig. 2C. Fig. S9. Uncropped immunoblots for Figs 3E and 5A. Fig. S10. Uncropped immunoblots for Fig. S2B. Fig. S11. Uncropped immunoblots for Fig. S2D. Fig. S12. Uncropped immunoblots for Fig. S4A,B. Fig. S13. Uncropped immunoblots for Fig. S5A. Fig. S14. Uncropped immunoblots for Fig. S5B. Fig. S15. Raw image data of representative wells from the clonogenic survival assay performed in U2OS cells. Fig. S16. Raw image data of representative wells from the clonogenic survival assay performed in HCT116 cells. [file MOL2-18-2179-s001.zip › mol213645-sup-0009-FigureS9.pdf]

Supplementary Figure 10.

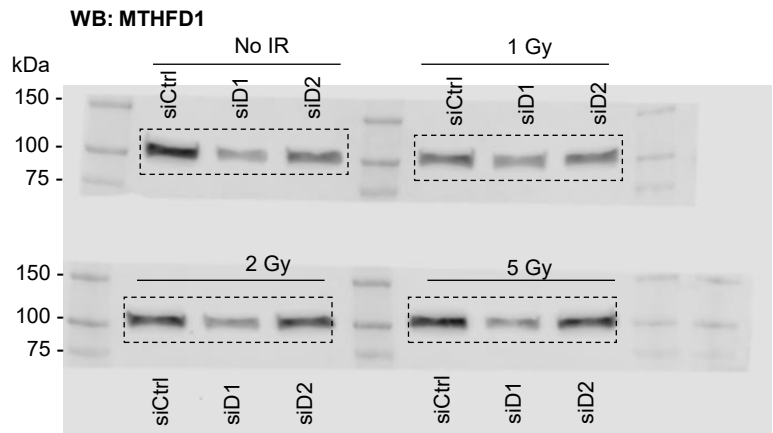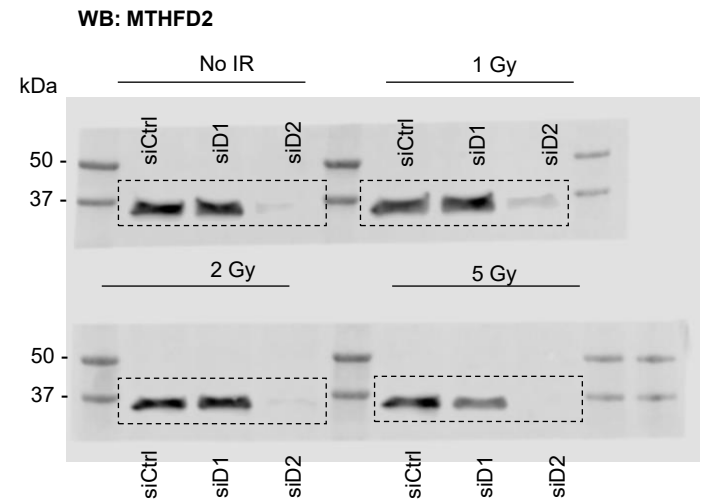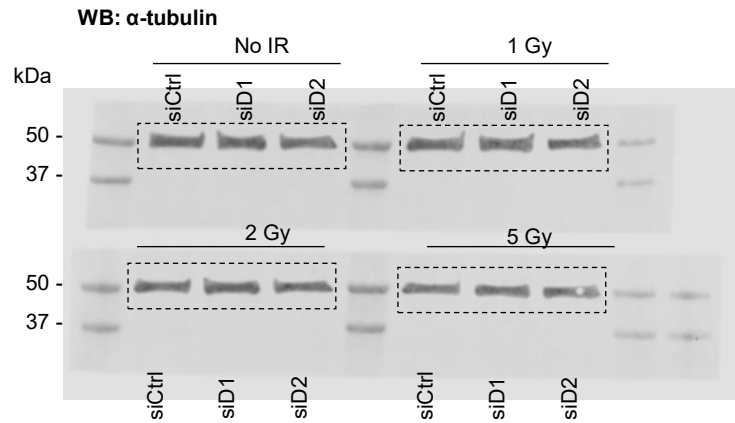

Supplement: Supplementary file 1 — Fig. S1. MTHFD2 but not MTHFD1 accumulates in the nucleus following IR treatment. Fig. S2. MTHFD2 silencing impairs cancer cell survival after irradiation. Fig. S3. Depletion of MTHFD2 hampers cell proliferation following irradiation. Fig. S4. MTHFD2 does not interact with ATM, DNA‐PK or RPA70. Fig. S5. MTHFD2 promotes DSB repair. Fig. S6. Uncropped immunoblots for Fig. 1A. Fig. S7. Uncropped immunoblots for Fig. 1C. Fig. S8. Uncropped immunoblots for Fig. 2C. Fig. S9. Uncropped immunoblots for Figs 3E and 5A. Fig. S10. Uncropped immunoblots for Fig. S2B. Fig. S11. Uncropped immunoblots for Fig. S2D. Fig. S12. Uncropped immunoblots for Fig. S4A,B. Fig. S13. Uncropped immunoblots for Fig. S5A. Fig. S14. Uncropped immunoblots for Fig. S5B. Fig. S15. Raw image data of representative wells from the clonogenic survival assay performed in U2OS cells. Fig. S16. Raw image data of representative wells from the clonogenic survival assay performed in HCT116 cells. [file MOL2-18-2179-s001.zip › mol213645-sup-0010-Figure10.pdf]

Supplementary Figure 11.

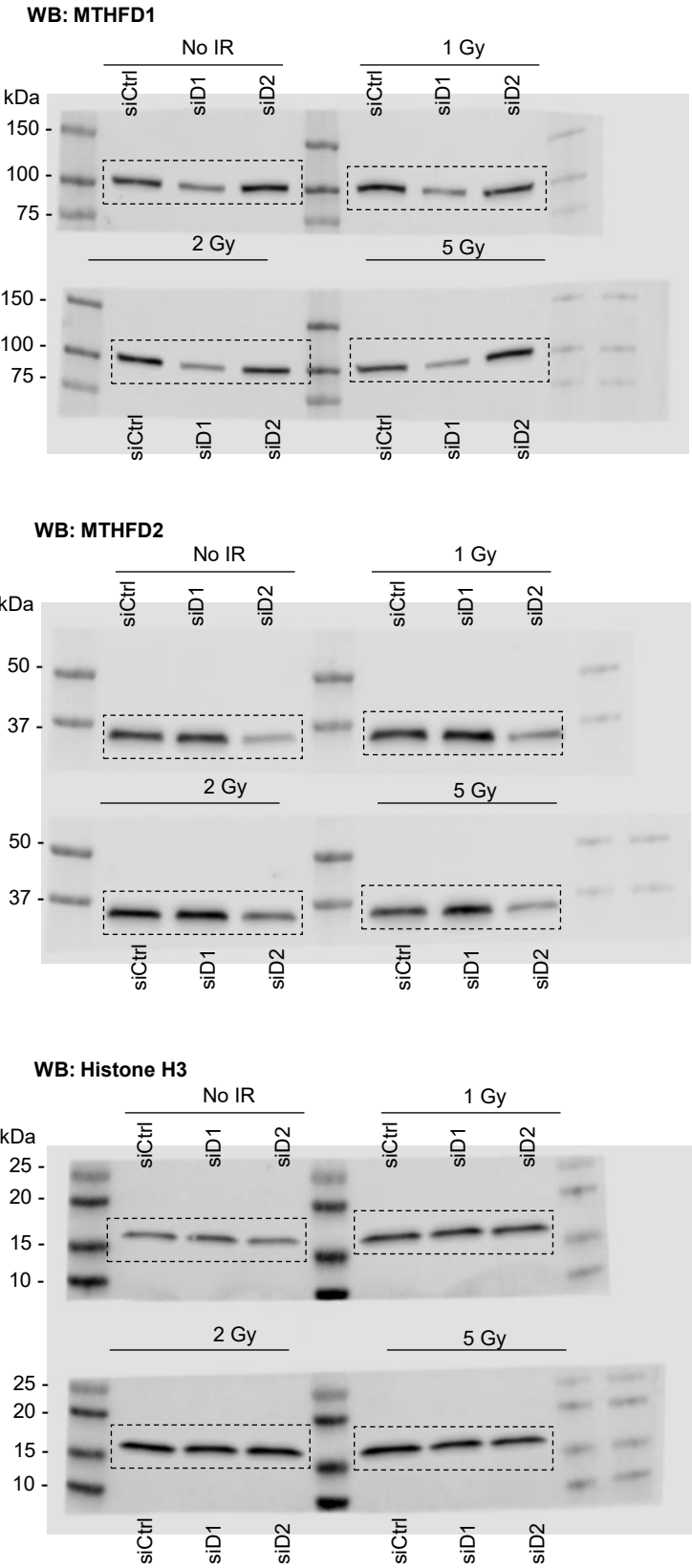

Supplement: Supplementary file 1 — Fig. S1. MTHFD2 but not MTHFD1 accumulates in the nucleus following IR treatment. Fig. S2. MTHFD2 silencing impairs cancer cell survival after irradiation. Fig. S3. Depletion of MTHFD2 hampers cell proliferation following irradiation. Fig. S4. MTHFD2 does not interact with ATM, DNA‐PK or RPA70. Fig. S5. MTHFD2 promotes DSB repair. Fig. S6. Uncropped immunoblots for Fig. 1A. Fig. S7. Uncropped immunoblots for Fig. 1C. Fig. S8. Uncropped immunoblots for Fig. 2C. Fig. S9. Uncropped immunoblots for Figs 3E and 5A. Fig. S10. Uncropped immunoblots for Fig. S2B. Fig. S11. Uncropped immunoblots for Fig. S2D. Fig. S12. Uncropped immunoblots for Fig. S4A,B. Fig. S13. Uncropped immunoblots for Fig. S5A. Fig. S14. Uncropped immunoblots for Fig. S5B. Fig. S15. Raw image data of representative wells from the clonogenic survival assay performed in U2OS cells. Fig. S16. Raw image data of representative wells from the clonogenic survival assay performed in HCT116 cells. [file MOL2-18-2179-s001.zip › mol213645-sup-0011-Figure11.pdf]

Supplementary Figure 12.

a

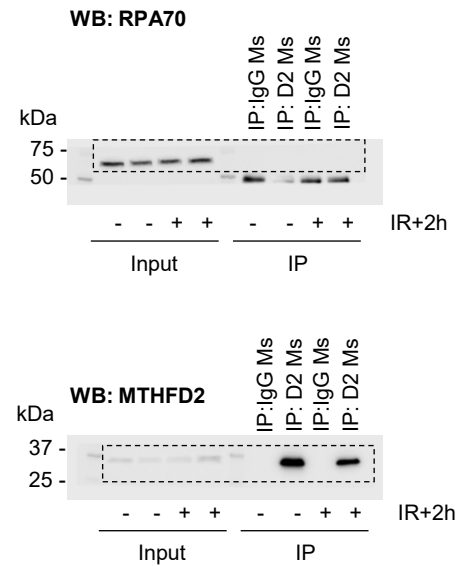

b

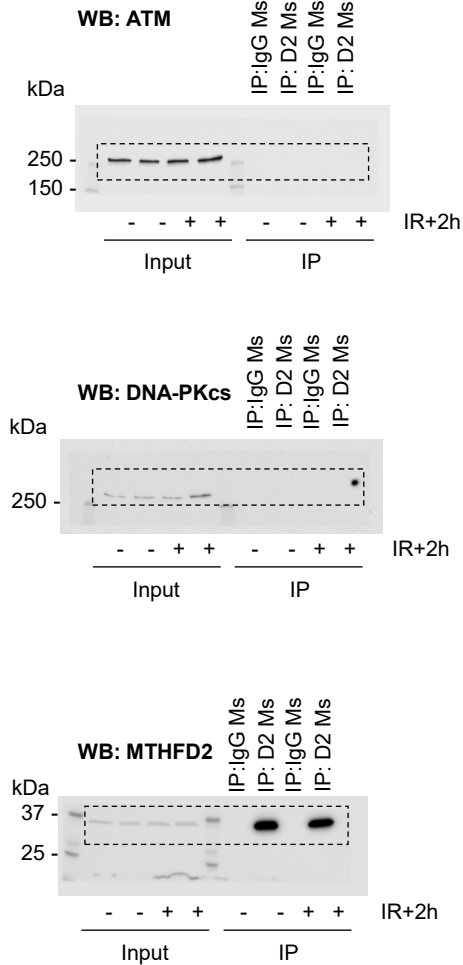

Supplement: Supplementary file 1 — Fig. S1. MTHFD2 but not MTHFD1 accumulates in the nucleus following IR treatment. Fig. S2. MTHFD2 silencing impairs cancer cell survival after irradiation. Fig. S3. Depletion of MTHFD2 hampers cell proliferation following irradiation. Fig. S4. MTHFD2 does not interact with ATM, DNA‐PK or RPA70. Fig. S5. MTHFD2 promotes DSB repair. Fig. S6. Uncropped immunoblots for Fig. 1A. Fig. S7. Uncropped immunoblots for Fig. 1C. Fig. S8. Uncropped immunoblots for Fig. 2C. Fig. S9. Uncropped immunoblots for Figs 3E and 5A. Fig. S10. Uncropped immunoblots for Fig. S2B. Fig. S11. Uncropped immunoblots for Fig. S2D. Fig. S12. Uncropped immunoblots for Fig. S4A,B. Fig. S13. Uncropped immunoblots for Fig. S5A. Fig. S14. Uncropped immunoblots for Fig. S5B. Fig. S15. Raw image data of representative wells from the clonogenic survival assay performed in U2OS cells. Fig. S16. Raw image data of representative wells from the clonogenic survival assay performed in HCT116 cells. [file MOL2-18-2179-s001.zip › mol213645-sup-0012-Figure12.pdf]

Supplementary Figure 13.

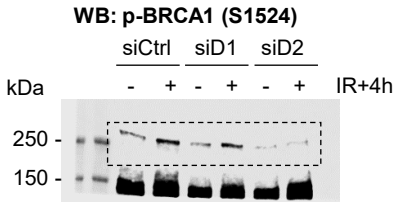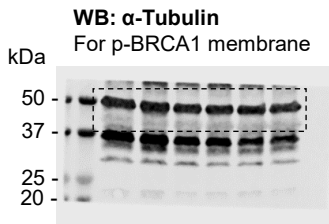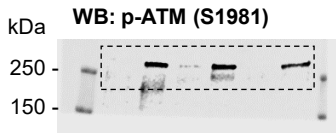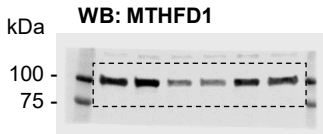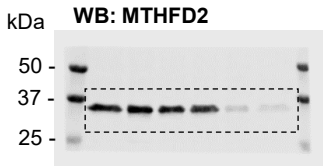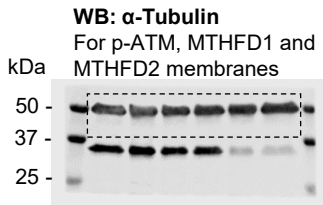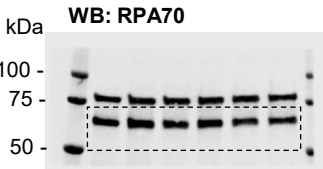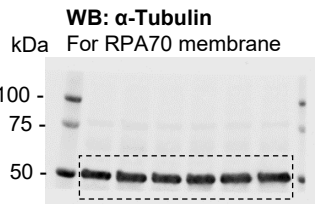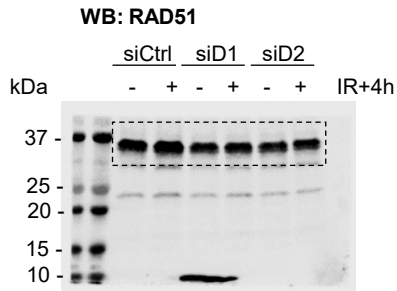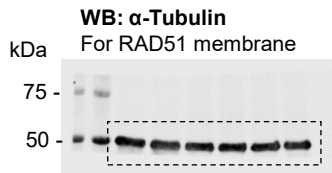

Supplement: Supplementary file 1 — Fig. S1. MTHFD2 but not MTHFD1 accumulates in the nucleus following IR treatment. Fig. S2. MTHFD2 silencing impairs cancer cell survival after irradiation. Fig. S3. Depletion of MTHFD2 hampers cell proliferation following irradiation. Fig. S4. MTHFD2 does not interact with ATM, DNA‐PK or RPA70. Fig. S5. MTHFD2 promotes DSB repair. Fig. S6. Uncropped immunoblots for Fig. 1A. Fig. S7. Uncropped immunoblots for Fig. 1C. Fig. S8. Uncropped immunoblots for Fig. 2C. Fig. S9. Uncropped immunoblots for Figs 3E and 5A. Fig. S10. Uncropped immunoblots for Fig. S2B. Fig. S11. Uncropped immunoblots for Fig. S2D. Fig. S12. Uncropped immunoblots for Fig. S4A,B. Fig. S13. Uncropped immunoblots for Fig. S5A. Fig. S14. Uncropped immunoblots for Fig. S5B. Fig. S15. Raw image data of representative wells from the clonogenic survival assay performed in U2OS cells. Fig. S16. Raw image data of representative wells from the clonogenic survival assay performed in HCT116 cells. [file MOL2-18-2179-s001.zip › mol213645-sup-0013-Figure13.pdf]

Supplementary Figure 14.

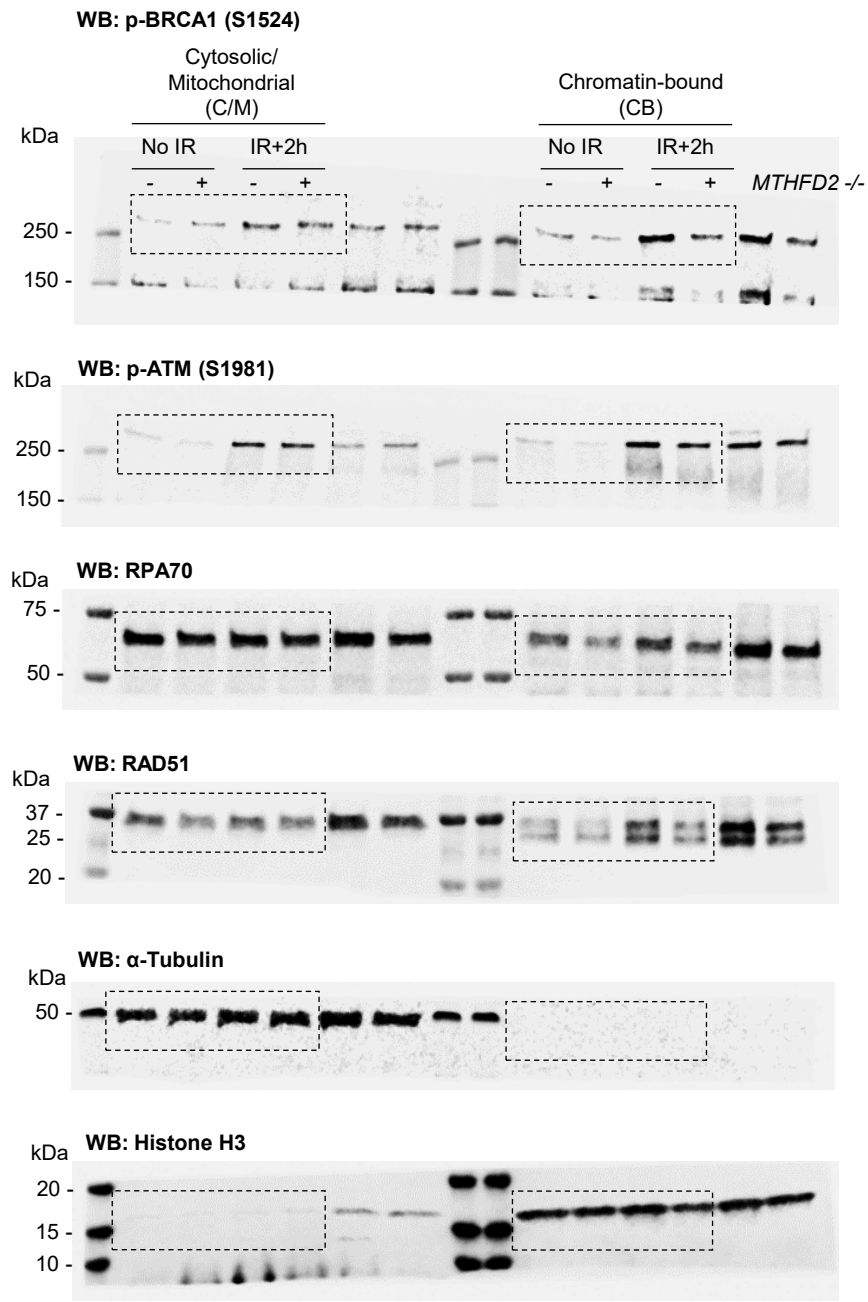

Supplement: Supplementary file 1 — Fig. S1. MTHFD2 but not MTHFD1 accumulates in the nucleus following IR treatment. Fig. S2. MTHFD2 silencing impairs cancer cell survival after irradiation. Fig. S3. Depletion of MTHFD2 hampers cell proliferation following irradiation. Fig. S4. MTHFD2 does not interact with ATM, DNA‐PK or RPA70. Fig. S5. MTHFD2 promotes DSB repair. Fig. S6. Uncropped immunoblots for Fig. 1A. Fig. S7. Uncropped immunoblots for Fig. 1C. Fig. S8. Uncropped immunoblots for Fig. 2C. Fig. S9. Uncropped immunoblots for Figs 3E and 5A. Fig. S10. Uncropped immunoblots for Fig. S2B. Fig. S11. Uncropped immunoblots for Fig. S2D. Fig. S12. Uncropped immunoblots for Fig. S4A,B. Fig. S13. Uncropped immunoblots for Fig. S5A. Fig. S14. Uncropped immunoblots for Fig. S5B. Fig. S15. Raw image data of representative wells from the clonogenic survival assay performed in U2OS cells. Fig. S16. Raw image data of representative wells from the clonogenic survival assay performed in HCT116 cells. [file MOL2-18-2179-s001.zip › mol213645-sup-0014-Figure14.pdf]

**Supplementary Figure 15.**

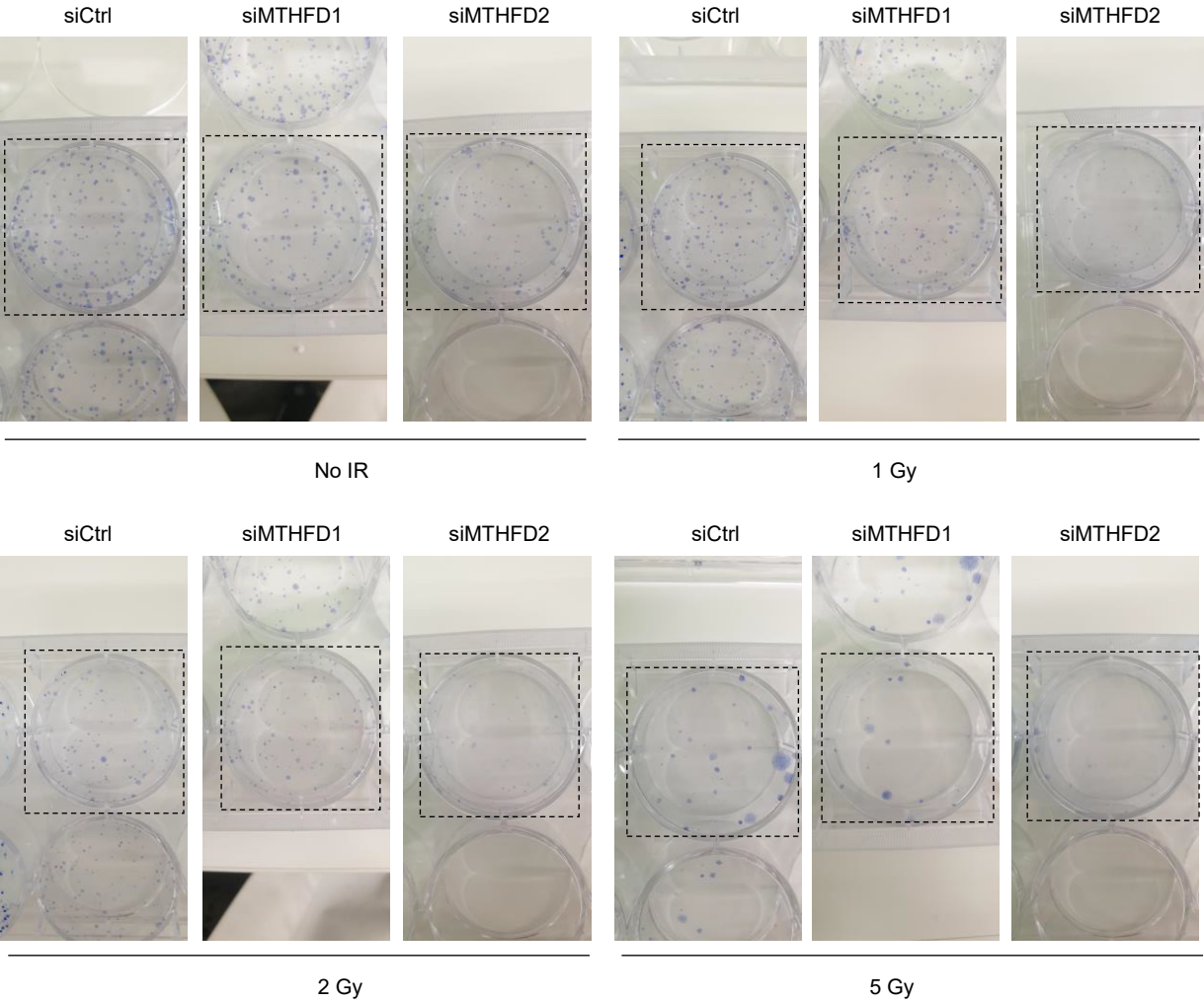

Supplement: Supplementary file 1 — Fig. S1. MTHFD2 but not MTHFD1 accumulates in the nucleus following IR treatment. Fig. S2. MTHFD2 silencing impairs cancer cell survival after irradiation. Fig. S3. Depletion of MTHFD2 hampers cell proliferation following irradiation. Fig. S4. MTHFD2 does not interact with ATM, DNA‐PK or RPA70. Fig. S5. MTHFD2 promotes DSB repair. Fig. S6. Uncropped immunoblots for Fig. 1A. Fig. S7. Uncropped immunoblots for Fig. 1C. Fig. S8. Uncropped immunoblots for Fig. 2C. Fig. S9. Uncropped immunoblots for Figs 3E and 5A. Fig. S10. Uncropped immunoblots for Fig. S2B. Fig. S11. Uncropped immunoblots for Fig. S2D. Fig. S12. Uncropped immunoblots for Fig. S4A,B. Fig. S13. Uncropped immunoblots for Fig. S5A. Fig. S14. Uncropped immunoblots for Fig. S5B. Fig. S15. Raw image data of representative wells from the clonogenic survival assay performed in U2OS cells. Fig. S16. Raw image data of representative wells from the clonogenic survival assay performed in HCT116 cells. [file MOL2-18-2179-s001.zip › mol213645-sup-0015-Figure15.pdf]

Supplementary Figure 16.

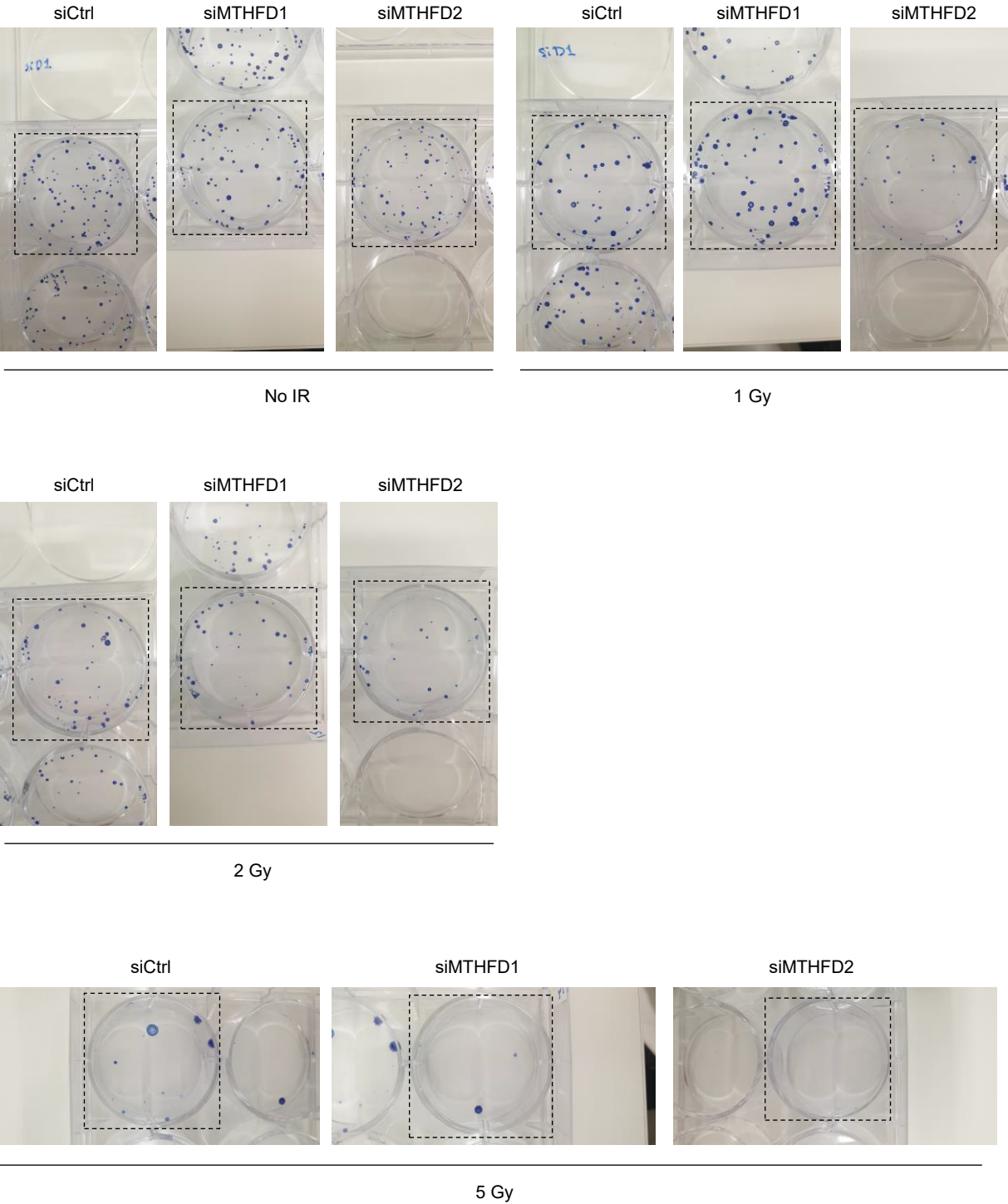

Supplement: Supplementary file 1 — Fig. S1. MTHFD2 but not MTHFD1 accumulates in the nucleus following IR treatment. Fig. S2. MTHFD2 silencing impairs cancer cell survival after irradiation. Fig. S3. Depletion of MTHFD2 hampers cell proliferation following irradiation. Fig. S4. MTHFD2 does not interact with ATM, DNA‐PK or RPA70. Fig. S5. MTHFD2 promotes DSB repair. Fig. S6. Uncropped immunoblots for Fig. 1A. Fig. S7. Uncropped immunoblots for Fig. 1C. Fig. S8. Uncropped immunoblots for Fig. 2C. Fig. S9. Uncropped immunoblots for Figs 3E and 5A. Fig. S10. Uncropped immunoblots for Fig. S2B. Fig. S11. Uncropped immunoblots for Fig. S2D. Fig. S12. Uncropped immunoblots for Fig. S4A,B. Fig. S13. Uncropped immunoblots for Fig. S5A. Fig. S14. Uncropped immunoblots for Fig. S5B. Fig. S15. Raw image data of representative wells from the clonogenic survival assay performed in U2OS cells. Fig. S16. Raw image data of representative wells from the clonogenic survival assay performed in HCT116 cells. [file MOL2-18-2179-s001.zip › mol213645-sup-0016-Figure16.pdf]
